# Comprehensive Characterization of the Viscoelastic Properties of Bovine Submaxillary Mucin (BSM) Hydrogels and the Effect of Additives

**DOI:** 10.1021/acs.biomac.4c00153

**Published:** 2024-06-04

**Authors:** Hanna Rulff, Robert F. Schmidt, Ling-Fang Wei, Kerstin Fentker, Yannic Kerkhoff, Philipp Mertins, Marcus A. Mall, Daniel Lauster, Michael Gradzielski

**Affiliations:** † Institute of Chemistry, Technische Universität Berlin, 10623 Berlin, Germany; ‡ Institute of Pharmacy, Freie Universität Berlin, 14195 Berlin, Germany; § Proteomics Platform, Max-Delbrück-Center for Molecular Medicine, 13125 Berlin, Germany; ∥ Research Center of Electron Microscopy, Institute of Chemistry and Biochemistry, Freie Universität Berlin, 14195 Berlin, Germany; ⊥ Institute of Chemistry and Biochemistry, Freie Universität Berlin, 14195 Berlin, Germany; # Berlin Institute of Health at Charite, Universitätsmedizin Berlin, 10178 Berlin, Germany; ∇ Department of Pediatric Respiratory Medicine, Immunology and Critical Care Medicine, Charite, Universitätsmedizin Berlin, 13353 Berlin, Germany; ○ German Centre for Lung Research (DZL), Associated Partner Site, 13353 Berlin, Germany

## Abstract

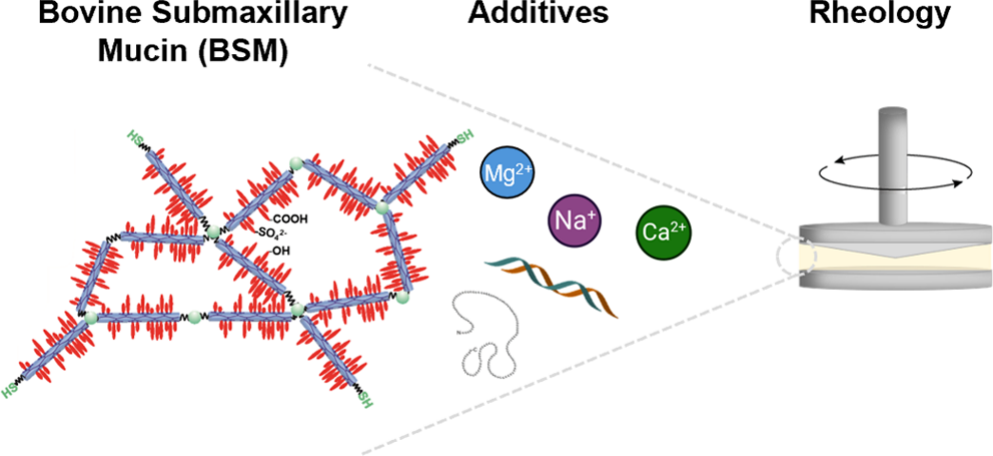

This study presents a comprehensive characterization of the viscoelastic and structural properties of bovine submaxillary mucin (BSM), which is widely used as a commercial source to conduct mucus-related research. We conducted concentration studies of BSM and examined the effects of various additives, NaCl, CaCl_2_, MgCl_2_, lysozyme, and DNA, on its rheological behavior. A notable connection between BSM concentration and viscoelastic properties was observed, particularly under varying ionic conditions. The rheological spectra could be well described by a fractional Kelvin–Voigt model with a minimum of model parameters. A detailed proteomics analysis provided insight into the protein, especially mucin composition within BSM, showing MUC19 as the main component. Cryo-scanning electron microscopy enabled the visualization of the porous BSM network structure. These investigations give us a more profound comprehension of the BSM properties, especially those pertaining to viscoelasticity, and how they are influenced by concentration and environmental conditions, aspects relevant to the field of mucus research.

## 1 Introduction

Mucus is an essential component for the mechanical and biological protection of many organisms, which covers epithelial surfaces. In humans, mucus acts as a protective barrier for mucosal surfaces.^1^ Mucus is a complex hydrogel constituted by more than 95% of water and (glyco)proteins, lipids, DNA, and salts.^2,3^ Mucus owes its diverse and proper functioning to the interaction of its components, the most important being mucin glycoproteins. One of the central mucus features is its hydrogel-like viscoelastic characteristic, which is essential to limit the diffusion of pathogenic particles and to allow efficient transport via cilia-supported transport (mucociliary clearance) in the airways^4^ or the peristaltic transport in the intestine. The hydrogel-gel-like characteristic of mucus is shaped by mucins.^3,5,6^ Mucins are high-molecular-weight macromolecules showing extensive glycosylation, which allows for water-binding capacity, and together with the mainly disordered protein backbone structure, is responsible for the hydrogel characteristics.^7−9^ Mucins form three-dimensional networks with a mesh-like structure, being responsible for the biophysical properties of mucus.^10−12^

Native human mucus is a complex and heterogeneous biomaterial, which makes the generation of reproducible data challenging due to its natural polymeric dispersity within and among individual hosts^3^ as well as the limited availability of mucus from human sources. Therefore, mucus from other sources possessing defined and reproducible properties and being available in larger quantities may provide suitable models for mucus research.^10,13^ Here, the viscoelastic properties are a common feature of different mucus types arising from varying sources.^13,14^ One commercially available mucus system is bovine submaxillary mucin (BSM) that is obtained by purification processes from extracts of fresh cattle submaxillary glands.^15^ However, BSM is not completely pure, and contains, besides large quantities of glycoproteins, nonmucin proteins (for example, bovine serum albumin (BSA)), DNA, lipids, and inorganic salts.^16^ BSM contains mucins^17^ which are similar to human respiratory mucins.^18,19^ They are known to confer viscoelastic properties to mucus and to be highly entangled polymers^20^ that are dominated by repeating structural motifs that react sensitively to changes in pH and concentration of Ca^2+^ ions.^21^ Accordingly, BSM can be considered as a model system for human mucus, having the advantage of being available in larger amounts of reproducible quality.

Such model systems are essential for gaining a fundamental understanding of the molecular origins of the viscoelastic properties of mucus and to learn how they might be affected by changes of the solvent conditions (e.g., ionic strength, salt, etc.) or the presence of other components such as proteins or DNA. This knowledge of the mechanical response of mucus is crucial for gaining a deepened understanding, for instance, of muco-obstructive lung diseases such as cystic fibrosis (CF).^6,22,23^ Due to altered viscous and elastic properties of sputum in different stages of disease and therapy, measuring the rheological properties turned out to be a potential biomarker.^14,24−26^ Noting this promising connection in mind, BSM has already been studied to some extent. Thornton et al. designed technologies for the detection and quantification of mucins, also for BSM,^18^ while other groups gave insights into the BSM genome.^19,27^ Lee et al. studied the behavior of BSM on hydrophobic surfaces observing the formation of elastic films,^28^ also by changing the experiment’s environmental conditions.^29^ BSM was also tested to produce mucin-based hydrogels showing physiologically relevant properties by cross-linking it with different cross-linking reagents.^13,14^ Furthermore, commercially available mucins like porcine gastric mucin (PGM) and BSM were subjected to characterization and comparison with human sputum by Lee et al. They recommend using BSM as the currently most suitable commercially available mucin source for replicating saliva based on its surface adsorption and lubrication properties.^30^ Feiler et al. studied the viscoelastic properties of BSM and BSA upon adsorption to surfaces and their potential for applications in biomaterial coating, showing the ability of BSM to form viscoelastic and more rigid layers.^31^ In a mixture of BSM with β-lactoglobulin, BSM was found to decrease the viscoelastic moduli, and thus the stability of the viscoelastic network studied here at an air–liquid interface.^32^

A study from Seller et al. gave insights into the gel formation of pig and sheep submaxillary mucins (PSM and SSM) influenced by their intact polymeric structure.^33^ The rheological behavior of commercially available PGM was subjected to a comparison to isolated natural porcine gastric mucus, indicating a more robust network formed by the natural mucus.^34^ Additionally, the influence of, e.g., ionic strength, pH, and concentration on the rheological properties of commercially available PGM as well as isolated porcine gastric and duodenal mucins were studied, showcasing its adaptable mechanical properties in different environments.^35,36^ Ribbeck et al. provided valuable insights into the complex and adaptable rheological properties of purified MUC5AC from fresh pig stomach scrapings, highlighting the influence of environmental factors such as pH, salt, and surfactants on their viscoelastic behavior.^37^

Despite comprehensive work on the rheological properties of mucins, this important class of hydrogels is far from being fully understood, which motivated us to provide a thorough characterization of the viscoelastic properties of BSM as a biological model material for mucus, such as respiratory mucus. We performed a comprehensive investigation of the rheological properties of BSM as a function of concentration, which we varied over a wide range from 10 to 140 g/L. It is noteworthy that the mucin concentration in healthy mucus is 20–50 g/L,^22,38^ whereas in CF patients, it can be approximately 5 times higher.^12,39^ In addition, we addressed effects arising from the presence of different salts, lysozyme as a model protein, and DNA. As the main findings, we could quantify how the viscous and elastic properties of BSM change as a function of concentration. The viscoelastic properties of BSM vary quite strongly upon addition of Ca^2+^ or Mg^2+^, while they are rather insensitive to the addition of Na^+^, lysozyme, or DNA. Another interesting finding is that the rheological data can be described very well by employing a fractional Kelvin–Voigt model, which apparently is well adapted to describe the rheological behavior of mucins.

## 2 Materials and Methods

### 2.1 Preparation of Mucus from Bovine Submaxillary Mucin

BSM (Mucin, Bovine Submaxillary Gland, Merck KGaA, Darmstadt, Germany, LOT 3776068, and LOT 3829388) was dissolved in Dulbecco’s phosphate-buffered saline (DPBS) without calcium and magnesium (containing 8 mg/mL sodium chloride) (Lot No. RNBJ1061, Sigma-Aldrich, Merck KGaA, Darmstadt, Germany) for 45 min at room temperature with a magnetic stirrer at the lowest speed (100 rotations per minute) to prevent the formation of bubbles. We prepared different concentrated BSM solutions with 10, 20, 60, 100, 140 mg/mL (= 1.0, 2.0, 6.0, 10.0, 14.0% w/v). pH measurements were performed directly after preparation using a microelectrode (InLab Micro, Mettler-Toledo GmbH, Gießen, Germany). After this, the rheology of the samples was measured.

### 2.2 Measurement of the Calcium and Sodium Content in BSM with Inductively Coupled Plasma–Optical Emission Spectrometry (ICP-OES)

For the BSM solutions of both batches (LOT 3776068 and LOT 3829388) the content of sodium, calcium, and magnesium was quantified by ICP-OES using a Varian ICP-OES 715 ES spectrometer. Details of the procedure are given in the Supporting Information (SI).

### 2.3 Detection of the DNA Content in BSM Solution

The DNA content determination protocol was adapted from published literature,^40^ and the DNA content was determined based on the reaction of 3,5-diaminobenzoic acid dihydrochloride (DABA; TCI Deutschland GmbH, Eschborn, Germany) and aldehyde group in DNA. The DNA reaction solution comprised 20% w/v DABA in Milli-Q water. 30 μL of BSM samples (LOT: 3678870) was mixed with 30 μL DNA detection solution and followed with incubation at 60 °C for 1 h. The reaction was quenched by the addition of 1 mL of 1.75 M HCl. Fluorescence intensity was measured with a Tecan M200 pro (Tecan Group Ltd., Männedorf, Switzerland) at excitation and emission wavelengths of 413 and 512 nm, respectively. The DNA concentration in BSM samples was calculated from the calibration curve which was generated with using known concentrations of DNA solution from salmon testes (single-stranded DNA from salmon testes, D9156, Sigma-Aldrich, Merck KGaA, Darmstadt, Germany).

### 2.4 Measurement of Protein Concentration in BSM Solution

The protein concentration determination procedure was followed with a Pierce BCA protein assay kit manual (Thermo Fisher Scientific, Rockford, IL, USA). A 25 μL portion of BSM samples in a transparent 96-well plate was mixed with 200 μL of working reagent, which is composed of 1.96% v/v of reagent B and 98.04% v/v reagent A. After incubation at 37 °C for 30 min, absorbance intensity was measured with a Tecan M200 pro (Tecan Group Ltd., Männedorf, Switzerland) at a wavelength of 562 nm. The protein concentration in BSM samples was calculated from the calibration curve, which was generated using known concentrations of bovine serum albumin (BSA) solution (Thermo Fisher Scientific, Rockford, IL, USA). The protein concentration was 85.55 mg/mL in a 100 mg/mL BSM solution.

### 2.5 Addition of Salts, DNA, and Lysozyme to a 100 mg/mL BSM Solution

To study the effect of different additives on the viscoelastic properties of BSM (LOT: 3829388), we prepared 100 mg/mL BSM solutions using the previously described method and added the following substances: Four different concentrations of sodium chloride (≥99.5%, S9888, Sigma-Aldrich, Merck KGaA, Darmstadt, Germany) 0.24, 0.38, 2.19, and 5.18 M in a 100 mg/mL BSM solution; nine different concentrations of calcium chloride (as calcium chloride dihydrate, ≥99.0%, C7902, Merck KGaA, Darmstadt, Germany) 5.25, 8.78, 13.95, 24.16, 51.37, 61.57, 78.58, 100.35, and 150.10 mM in a 100 mg/mL BSM solution, which was prepared with 20 mM HEPES buffer 2-[4-(2-hydroxyethyl)piperazin-1-yl]ethanesulfonic acid ( ≥99.5%, H3375, Sigma-Aldrich, Merck KGaA, Darmstadt, Germany) as calcium chloride is not soluble in DPBS-buffer; and eight different concentrations of magnesium chloride (magnesium chloride anhydrous, ≥98.0%, CAS-Nr.: 7786-30-3, Avantor, VWR International GmbH, Darmstadt, Germany, ) 4.97, 8.75, 13.40, 24.00, 49.40, 76.40, 101.40, and 151.40 mM in a 100 mg/mL BSM solution with 20 mM HEPES buffer as well; see the SI for detailed procedure.

Four different concentrations of desoxyribonucleic acid (DNA) from salmon testes (Deoxyribonucleic acid sodium from salmon testes, D1626, Sigma-Aldrich) 5 mg/mL, 10 mg/mL, 15 and 20 mg/mL were present in a 100 mg/mL BSM solution. Lysozyme (Muramidase from hen egg white, ≥95.0%, Roche Diagnostics GmbH, Mannheim, Germany) was added in concentrations of 1.0, 2.0, and 10.0 mg/mL in a 100 mg/mL BSM solution. All solutions were well stirred for 2 h and stored in the fridge for 24 h. Afterward, they were let to adjust to room temperature, the pH values were measured as stated above, and are given in the Results and Discussion section. Then, the rheological measurements were performed.

### 2.6 Rheology

All rheology measurements were performed with an Anton-Paar MCR 502 WESP (Anton-Paar Germany GmbH, Ostfildern-Scharnhausen, Germany) temperature-controlled rheometer by using a cone–plate geometry with a cone angle of 1° and a cone diameter of 25 mm. The gap was set to 48 μm. The sample was transferred onto the lower static plate of the rheometer with a nonelectrostatic spatula and the upper cone was lowered slowly. The measurements were performed at 25 °C under saturated atmosphere using a solvent trap whose reservoir was filled with 2 mL of Milli-Q water to avoid evaporation effects. After reaching a steady temperature of 25 °C and waiting for 5 min, measurements were started with an amplitude sweep.

The linear viscoelastic (LVE) region and the critical deformation were determined by an amplitude sweep performed at a constant frequency of 1 Hz (6.28 rad/s) and covering a range of strain amplitudes between 0.01% and 100%. The LVE region represents the range of amplitude strain values over which the viscoelastic parameters are independent of the applied forces.^41^ Based on the amplitude sweeps (see Figure S3) the strain amplitude of the oscillatory experiments was set to 1% and the frequency was varied between 0.05 Hz (0.314 rad/s) and 50 Hz (314 rad/s) utilizing the same sample aliquot. The frequency sweep was first performed from low to high and thereafter from high to low frequencies and they were interpreted regarding the viscoelastic material properties in terms of the storage (*G*′) and the loss (*G*″) modulus, or alternatively via the phase angle δ (°) and the complex viscosity η* (Pa·s), which are directly related to each other via
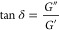
1
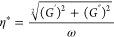
2A fully elastic material shows a phase angle of δ = 0°, while for fully viscous behavior, a phase angle δ = 90° is observed. According to eq 1 for δ < 45°, the elastic properties dominate, while for predominant viscous behavior, it is δ > 45°. The complex viscosity is another parameter to formulate the viscoelastic properties of a material, which can be compared to the shear viscosity. The shear viscosity was studied with a new aliquot of each sample in constant shear experiments, in which we varied the shear rate from 0.1 to 1000 s^–1^, going from low to high and afterward from high to low shear rates, in order to check for potential hysteresis effects. Data analysis was performed with GraphPad Prism version 10.1.2 (GraphPad Software, San Diego, CA) and Origin, version 2021b (OriginLab Corporation, Northampton, MA).

### 2.7 Fitting of Rheological Data

The frequency-dependent moduli were fitted with a fractional Kelvin–Voigt model (FKVM).

Classical viscoelastic models are based on springs and dashpots, characterized by an elastic modulus, *G*, and a viscosity, η, respectively. Fractional viscoelastic models employ a mechanical element known as the spring-pot, which has intermediate properties between a spring and a dashpot, and its constitutive equation employs a fractional derivative of the order 0 < α < 1. For α = 0, a regular spring is retrieved, and for α = 1, a regular dashpot is retrieved. The associated material property, η_α_, referred to as a *quasi-property*, has units of Pa·s^α^ and therefore lacks direct physical meaning, but can be associated with the firmness of a material.^42^ Fractional viscoelastic models are typically very good at describing power law rheological behavior, which occurs in materials that exhibit a broad range of relevant microstructural length and time scales,^43^ as is the case for many biological systems.^44−47^ More details on fractional viscoelastic models can be found elsewhere.^48,49^ Here, we employ a fractional Kelvin–Voigt model (FKVM), consisting of a spring with elastic modulus *G* and a spring-pot with quasi-property η_α_, arranged in parallel. *G*′ and *G*″ for the FKVM are given by
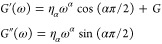
3At low frequencies, *G*′ dominates and approaches the elastic modulus *G*, while at high frequencies, both *G*′ and *G*″ follow the power law ∼ ω^α^.

**Scheme 1:**
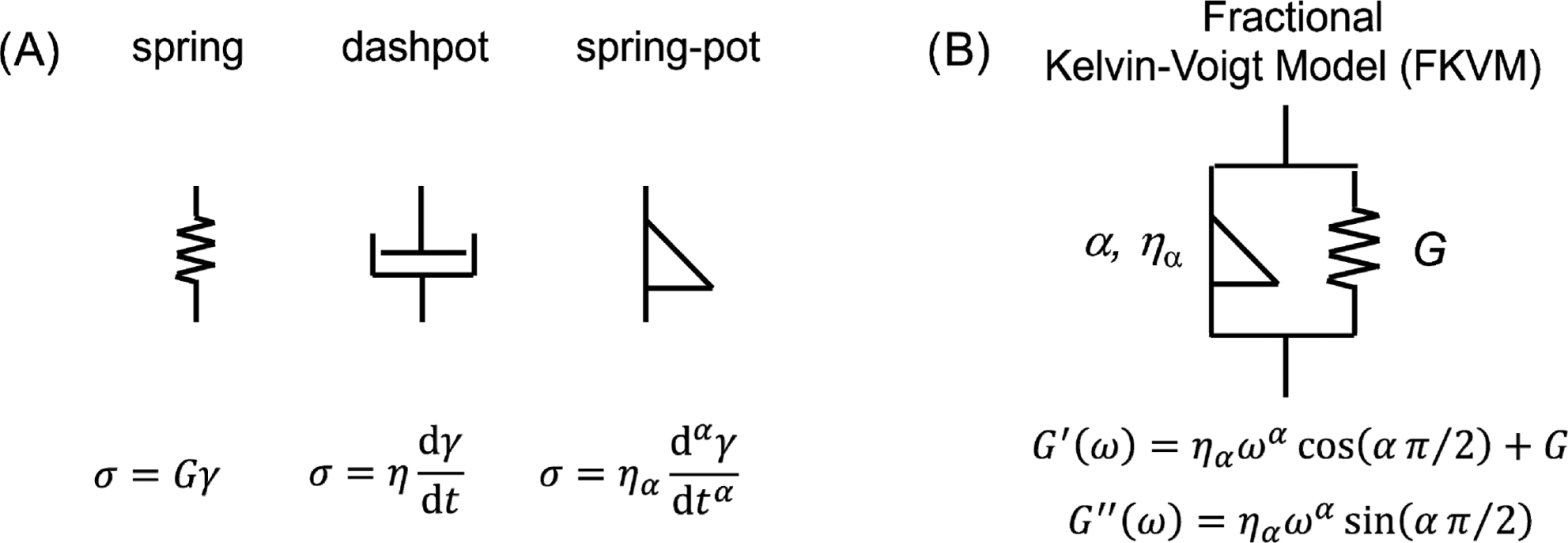
Viscoelastic Models Constitutive equations for (A) the spring, dashpot, and spring-pot model elements as well as (B) the fractional Kelvin–Voigt model; σ = stress, γ = strain.

A graphical representation of the FKVM is shown below (Scheme 1).

Fitting of the frequency-dependent moduli *G*′ and *G*″ was performed in Python using the lmfit package, which employs a nonlinear least-squares optimization routine.^50^ Model functions were written to return a 1D array containing *G*′ and *G*″, meaning both moduli are fitted at the same time. The minimized quantity is defined as
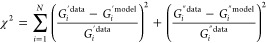
4to ensure all data points are considered equally. At high frequencies, there can be artifacts caused by sample and instrument inertia.^51^ For this reason, only angular frequencies ω < 50 rad/s = 7.96 Hz were considered for the fit.

### 2.8 Cryo-Scanning Electron Microscopy (cryo-SEM)

A small drop of a 100 mg/mL BSM solution of each batch was plunged into nitrogen slush at atmospheric pressure, freeze-fractured at −180 °C, etched for 60 s at −98 °C, and sputtered with platinum in a Gatan Alto 2500 cryo-preparation chamber. After transfer into the S-4800 cryo-SEM instrument (Hitachi, Tokyo, Japan), the morphology of the BSM was evaluated at an acceleration voltage of 2.0 kV.

For the quantitative SEM evaluation, 10 images of each batch of BSM solution were included in the analysis. The included images were taken at magnifications of 20.000×, 40.000×, 80.000×, and 100.000×. The SEM images were smoothed by applying a Gaussian filter with a sigma radius of 1 pixel. Images were automatically binarized by an Otsu threshold^52^ to separate them into the fiber network (bright) and pores (dark). The pores were analyzed with Fiji’s Particle Analyzer.^53^ Holes on the edges of the images were excluded from analysis to avoid artifacts. The maximal and minimal Feret diameter^54−56^ of each pore was automatically quantified and manually verified. The distribution of minimal Feret diameters of all pores per sample was visualized in a violin plot with the mean value and the single standard deviation indicated as lines within the violin plot. Details of the procedure are given in the SI (Figure S1).

### 2.9 Proteomic Measurements

BSM (LOT: 3829388) was dissolved in PBS at a concentration of 4 mg/mL. Three aliquots at 100 μg of mucin were reduced and alkylated in SDS buffer (2% SDS, 50 mM Tris-HCl pH 8, 0.5 mM EDTA, 75 mM NaCl, 10 mM DTT, 40 mM CAA final concentration) by incubating for 10 min at 95 °C. After cooling down, bezonase (10.25 U, Merck KGaA, Darmstadt, Germany) was added and samples were incubated for 15 min at room temperature. Subsequently, single-pot, solid-phase-enhanced sample preparation (SP3 cleanup) was performed.^57^ N-glycosylation was removed by a 1 h incubation with PNGase F (500 U, New England Biolabs GmbH, Frankfurt am Main, Germany) and following digest with sequence-grade trypsin (Promega GmbH, Walldorf, Germany) and lysyl endopeptidase (LysC, FUJIFILM Wako Pure Chemical Corporation) at a 1:50 enzyme:substrate ratio overnight at 37 °C. The digest was stopped by adding trifluoroacetic acid to a final concentration of 1%. The supernatant was collected, and peptides desalted using C18 stage tips.^58^ Peptides were reconstituted in 3% acetonitrile with 0.1% formic acid. 0.4 μg of peptides were separated on an EASY-nLC 1200 System on a reversed-phase column (20 cm fritless silica microcolumns with an inner diameter of 75 μm, in-house packed with ReproSil-Pur C18-AQ 1.9 μm resin (Dr. Maisch GmbH, Ammerbuch-Entringen, Germany)) using a 98 min gradient with a 250 nL/min flow rate of increasing Buffer B (90% ACN, 0.1% FA) concentration (from 2 to 60%).

Separated peptides were analyzed on an Orbitrap Q Exactive HF-X mass spectrometer (Thermo Fisher Scientific, Rockford, IL, USA) running on data-dependent acquisition (DDA) mode with a 60 k MS1 resolution, AGC target of 3 × 10^6^ ions, and a maximum injection time of 10 ms, choosing the top 20 ions for MS2 scans with 15 K resolution, AGC target of 1 × 10^5^ ions, and maximum injection time of 22 ms.

Database search was performed using MaxQuant (Ver. 2.0.3.0) using UniProt database for bovine proteins (downloaded 2022-09). Oxidation (M), N-terminal acetylation, and deamidation (N, Q) were set as variable modifications, and carbamidomethyl (C) was set as fixed modification. Match between runs, label-free quantification, and iBAQ algorithms were applied. Data are available via ProteomeXchange with the identifier PXD048381.

Downstream analysis was done in R (V 4.2.2). Proteins were filtered for “reverse” and “only-identified by site”. Proteins identified by the contaminants list were removed if they did not originate from the current bovine or cattle databases. Only proteins that were quantified in all of the replicates were considered for further analysis.

## 3 Results and Discussion

### 3.1 Comparison of Two Different Batches of Pure BSM Solution

First, we wanted to determine to which extent data obtained from rheological characterization may depend on the choice of BSM batch, as a batch-to-batch variability was an issue noted in the literature for commercially available mucus systems.^19,27,30,59,60^ Therefore, we used different batches of BSM, provided by Merck KgaA, prepared by the method of Nisizawa and Pigman,^15^ and indicated by different LOT numbers.

**Figure 1:**
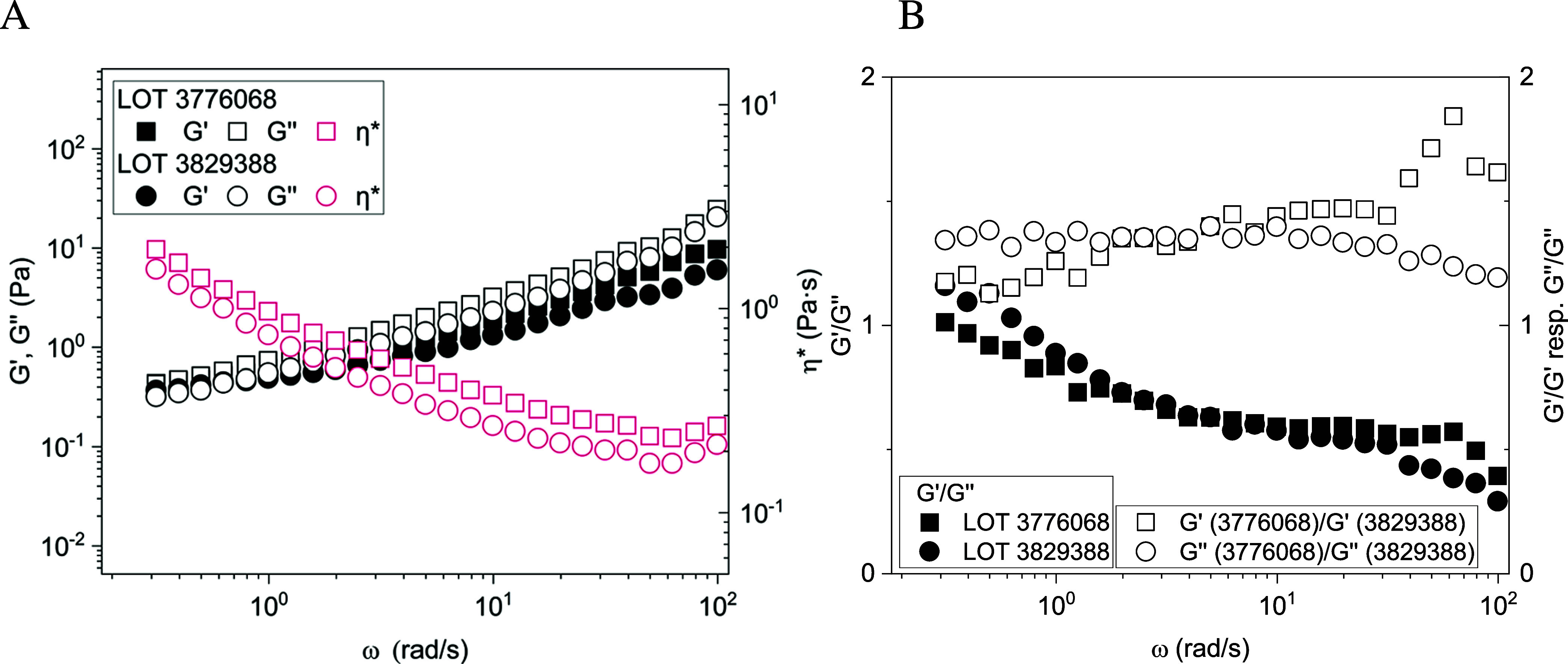
Viscoelastic properties of two different batches of BSM at pH 7.4. (A) Storage modulus *G*′ (Pa) and loss modulus *G*″ (Pa) as well as complex viscosity η* (Pa·s) of 100 mg/mL BSM solutions were measured as a function of angular frequency (rad/s) at 25 °C (data are shown as mean values of *n* = 3 measurements). (B) Ratio of *G*′/*G*″ for the given two batches, as well as ratio of *G*′/*G*′ and *G*″/*G*″ for the two different batches as a function of angular frequency (rad/s).

**Figure 2:**
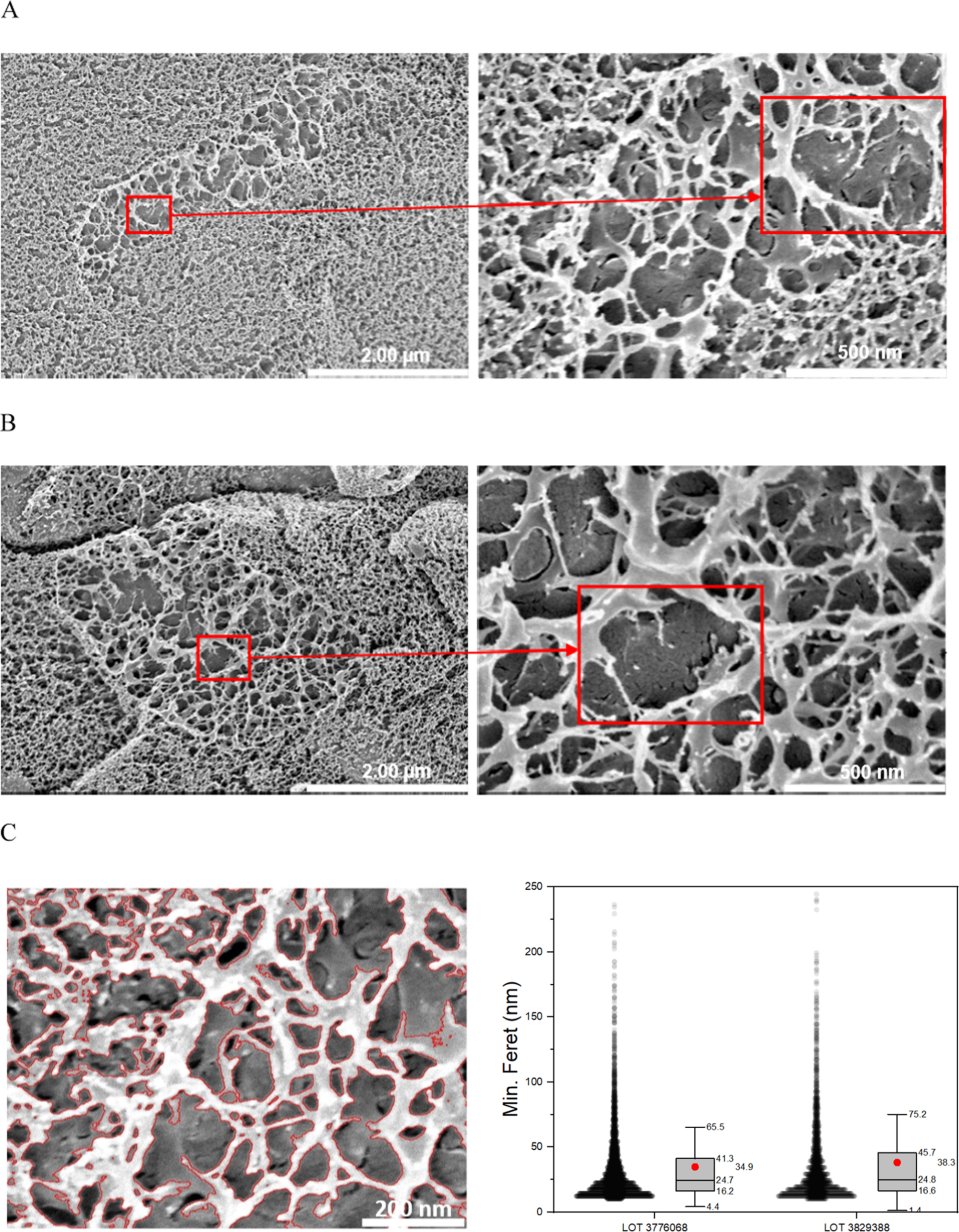
Exemplary SEM images. Cryogenic-scanning electron micrographs (cryo-SEM) at 20.000 (left) and 80.000 (right) × magnification of a 100 mg/mL BSM solution: (A) Batch-Nr. LOT 3776068 and (B) Batch-Nr. LOT 3829388. (C) Structured analysis of SEM images. Detected pores are outlined in red on original image crop of Batch-Nr. LOT 3776068 (left). Minimal Feret diameter (nm) of all pores per sample of each batch of 100 mg/mL BSM solution (right). All pore diameters are shown as individual dots, and the distribution is summarized with a boxplot showing the mean as red dot, the median as black line, the 25 and 75% intervals as box edges, and the positive single standard deviation as whisker.

From each batch, 100 mg/mL BSM solutions in DPBS were prepared and underwent oscillatory rheological shear measurement. For both BSM batches the loss modulus *G*″ was similarly larger than the storage modulus *G*′ within the investigated frequency range and *G*′ and *G*″ were increasing slowly with increasing frequency (Figure 1A). Notably, at lower frequencies, *G*′ became relatively more prominent, as seen from the ratio *G*′/*G*″ (Figure 1B), a comportment that is rather uncommon for viscoelastic soft matter systems. This type of rheological behavior clearly indicated gel-like properties in the investigated frequency range but with a distinct viscous component. However, as seen by the rather low values of the moduli in the range of 0.5 to 20 Pa these are rather soft gels. The absolute values differed systematically by about 10–50% (Figure 1B), which means that the selection of a given batch did not largely change the behavior but certainly had a non-negligible effect on the absolute viscoelastic values. However, when looking at the ratio *G*′/*G*″ (Figure 1B) the differences became very small, which means that the absolute values of *G*′ and *G*″ depend somewhat on the batch but the relative viscoelastic properties almost not. This similarity of the viscoelastic properties is also reflected in the mesh sizes (shown as the min Feret diameter [nm] in Figure 2C) obtained from a structural analysis of the cryo-SEM images.

### 3.2 Cryo-SEM Images Visualize BSM Network

For a further investigation of the network structure of BSM on a microscopic scale, cryo-scanning electron microscopy (cryo-SEM) experiments were performed at different magnifications for the two BSM batches, in each case a homogeneous solution with a concentration of 100 mg/mL BSM dissolved in DPBS.

The images allowed the visualization of the classical web-like structure of mucus.^30,59^ The complexity and flexibility of the mucus network and the interaction of larger and smaller mesh sizes could be identified. For both batches, areas with very small pores as well as areas with large pores were detected, i.e., a substantial structural inhomogeneity. These areas overlapped and were stacked on each other which is particularly well visible in Figure 2B; thus, forming 3D structures that can be imagined from a 2D representation such as SEM. The mucus network appeared very finely and visibly structured. There seemed to be no contamination with, for example, bacteria or cell debris. The largest visible mesh in the images was identified (20.000× magnification) and enlarged (80.000× magnification). It had a size of not more than 500 nm for both BSM batches (red boxes). More images are shown in SI Figure S2 that confirm the general validity of the structures depicted in Figure 2.

Overall, the network structure, including the pores and pore size distribution in the mucus appeared heterogeneous^61^ This goes in line with the pore size results from (cryo-) SEM studies from i.e., human airway mucus^62−64^ or porcine and canine gastric mucus collections^55,65,66^ Thus, BSM shows an internal network comparable to other mucus systems. The cryo-SEM preparation process was examined for its potential impact on mucus structure.^61,63,67^ The pore size distribution of the examined BSM was found to be mainly between 10 and 80 nm (Figure 2C). This is in the expected size range based on nanoparticle diffusion experiments in native mucus and substantially smaller than the reported pore sizes of artificial pores (micrometer range), which can be caused by the cryogenic preparation process.^63^ The cryo-SEM images of the BSM displayed structures and pore size distributions that were similar to those of mucus previously analyzed from various sources.

The pore size descriptor used was Feret’s minimum diameter, which refers to the shortest distance between two parallel tangents of a pore.^54−56^ This parameter is widely used to describe the sizes and distribution of pores in SEM mucus samples. The analysis showed no significant differences between the two BSM batches in terms of their pore size. The mean values of the two batches were 34.9 and 38.3 nm and therefore only approximately 3 nm apart. The distributions of pore sizes were also similar. Accordingly, one can conclude that the mucin hydrogel structure depended only a little on the choice of the BSM batch.

### 3.3 Concentration Dependence and the Effect of Additives on the Viscoelastic Properties of BSM Solutions

#### 3.3.1 Concentration Dependence

A central parameter that determines the viscoelasticity of hydrogels is the concentration of the network-forming material, and therefore we performed rheological measurements for five different concentrations: 10, 20, 60, 100, and 140 mg/mL in DPBS, all having the same pH value of 7.3, using the same batch of BSM (LOT: 38293889).

**Figure 3:**
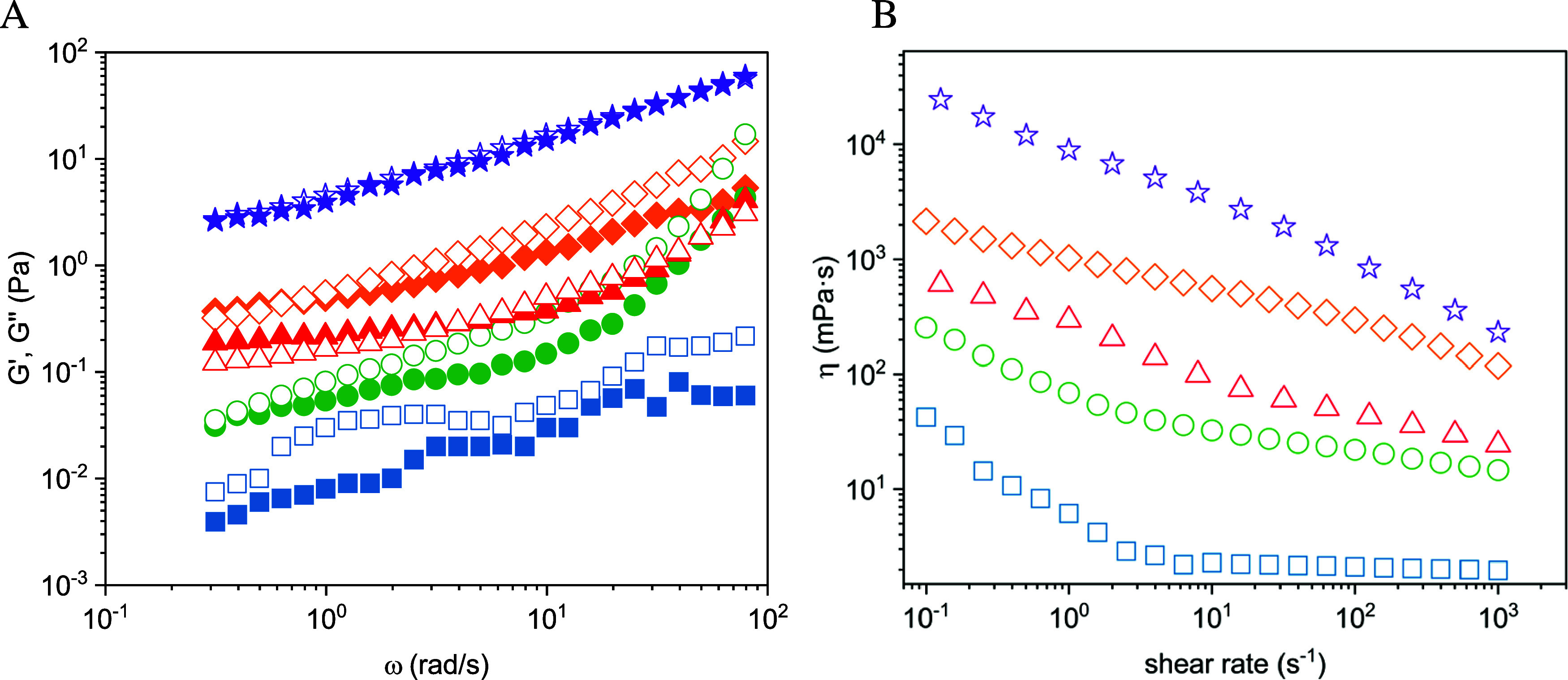
Viscoelastic moduli and steady shear viscosity of different concentrated BSM solutions. (A) Storage modulus *G*′ (Pa) (filled symbols) and loss modulus *G*″ (Pa) (open symbols) as a function of angular frequency for BSM solutions with different concentrations (deformation: 1%). (B) With a new aliquot of each solution, steady shear viscosity measurements as a function of shear rate were performed. The symbols indicate 10 (blue squares), 20 (green circles), 60 (red rectangles), 100 (orange rectangles), and 140 mg/mL (purple stars). Data shown are mean values of *n* = 3 measurements at each concentration. Individual values are shown in Table S1.

As shown in Figure 3A, we observed an increase of the viscoelastic moduli *G*′ and *G*″ over the investigated frequency range, this increase being clearly most marked for the more concentrated BSM solutions (60, 100, and 140 mg/mL) compared to the less concentrated ones (10 and 20 mg/mL). For the higher concentrations, significantly higher values for *G*′ and *G*″ were observed; for instance, *G*′ and *G*″ were around 2 orders of magnitude higher when comparing 100 and 10 mg/mL BSM solutions (Figure 3A). In general, the viscous component *G*″ was larger than the elastic component *G*′ over the investigated frequency range. The ratio *G*′/*G*″ slightly increased with increasing concentration (see Figure S4A), which means that the relative elastic properties of the hydrogels get larger.

In muco-obstructive lung diseases, such a relatively large increase in the viscoelastic properties caused here by increased mucin concentration is associated with reduced transportability of mucus through the cilia and thus impaired mucus clearance.^22^ Interestingly, a dominance of *G*″ over *G*′ can be observed in BSM, particularly at concentrations of 20 and 100 mg/mL. In contrast, sputum from patients with CF exhibits the opposite case, with a dominance of *G*′ over *G*″.^26^

**Figure 4:**
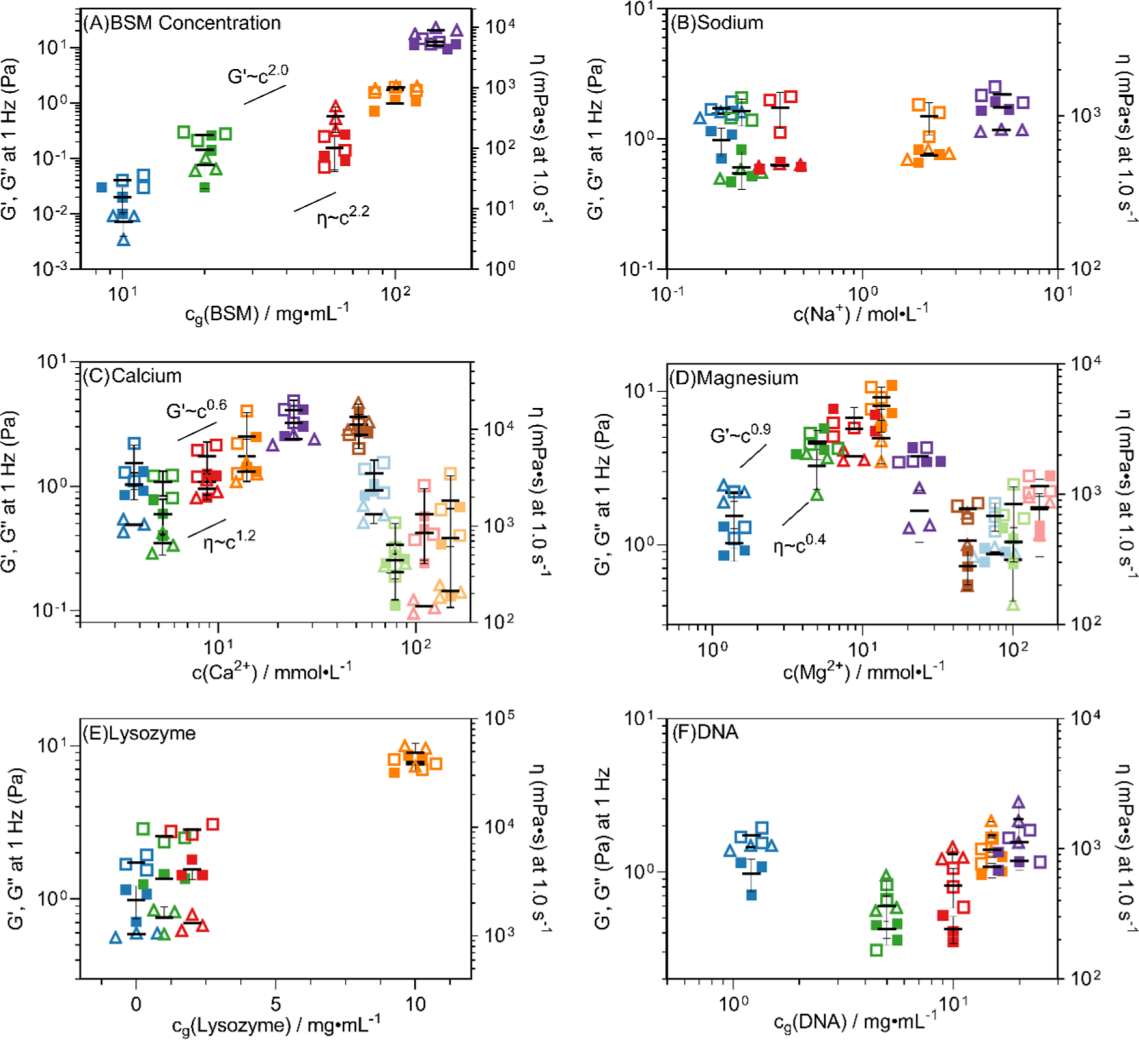
Viscoelastic properties of BSM solutions in different concentrations and additives. Storage modulus *G*′ (Pa) (filled squares) and loss modulus *G*″ (Pa) (open squares) at a representative frequency of 1 Hz (6.28 rad/s) are shown on the left *y*-axis, and shear viscosity η (mPa·s) (open triangles) at a representative shear rate of 1.0 s^–1^ is shown on the right *y*-axis, both depending on concentrations. Data are shown as individual values from the *n* = 3 repeated measurements, mean (horizontal line) and standard deviation (error bars). Individual values are shown in Table S1. (A) Concentration studies of BSM solutions: 10, 20, 60, 100, and 140 mg/mL (see Figures 3, S4, and S5). (B) Effect of added NaCl to a 100 mg/mL BSM solution: 0.19 M (baseline), 0.24, 0.38, 2.19, and 5.18 M Na^+^. (C) Effect of added CaCl_2_ to a 100 mg/mL BSM solution: 3.8 (baseline), 5.3, 8.78, 14.0, 24.2, 51.4, 61.6, 78.6, 100.4, and 150.0 mM Ca^2+^ (see Figure S7). (D) Effect of added MgCl_2_ to a 100 mg/mL solution: 1.4 (baseline), 5.0, 8.8, 13.4, 24.0, 49.4, 76.4, 101.4, and 151.4 mM Mg^2+^ (see Figure S8). (E) Effect of added lysozyme to a 100 mg/mL BSM solution: 0.0, 1.0, 2.0, and 10.0 mg/mL Lysozyme (see Figure S9). (F) Effect of added DNA to a 100 mg/mL BSM solution: 1.21 (baseline), 5.0, 10.0, 15.0, and 20 mg/mL DNA (see Figure S10). In cases in which a power law scaling of the storage modulus or the shear viscosity was observed, the scaling exponents are indicated.

A comparison of the values of *G*′ and *G*″ relative to the sample with 100 mg/mL BSM revealed an almost identical relative increase as a function of the frequency (Figure S4B,C). For a more straightforward comparison, Figure 4A illustrates *G*′ and *G*″ as functions of concentration at a frequency of 1.0 Hz. A power law increase with an exponent of ∼2.0 was observed, which is very similar to a scaling law in other viscoelastic hydrogels, such as Xanthan-Al (III) gels^68^ or chemically cross-linked poly(vinyl alcohol) near the gelation threshold.^69^ Theoretical work on the viscoelastic properties of entangled polymers predicts a power law with an exponent 2.0–2.3 for the plateau modulus *G*_0_.^70^ Apparently, the BSM behaves in agreement with this model of entangled polymers.

Steady shear viscosity measurements showed a very sharp increase in viscosity for shear rates below 10^–3^ s^–1^ (Figure S5A) and an effective divergence. This indicates that the samples have a finite yield stress but with a rather low value of ∼1 mPa, as shown in Figure S5B, and a rather low critical shear rate of 10^–4^–10^–3^ s^–1^. This is an uncommon behavior for physically entangled hydrogels, which normally show a finite maximum structural relaxation time. However, it corresponds well with the fact that *G*′ does not decrease strongly at low frequencies (Figure 3A). The origin of this behavior may be attributed to the fact that entanglements of the long mucin chains with their bulky side chains may take very long to resolve. In addition, S–S bonds constitute permanent cross-links which may contribute to this behavior. It should be noted here that the yielding behavior of pedal mucus has been the subject of extensive study.^71^ For higher shear rates, all BSM solutions exhibited shear thinning behavior, which was becoming somewhat more pronounced with increasing concentration (Figure 3B). Interestingly the shear rate dependence at shear rates above 10^–3^ s^–1^ followed a power law with a rather low exponent of 0.2–0.25 (Figure S5A), which indicates that the BSM solution would not quickly lose its viscous properties, even at rather high shear rates. This may also be important for a good functioning in biological systems.

The viscosity values increased systematically with increasing concentration. For comparison, Figure 4A shows the effective viscosity taken at a shear rate of 1.0 s^–1^. It increases with a power law with an exponent of ∼2.2.

Once a certain concentration has been surpassed one typically observes a power law increase of the viscoelastic moduli and the shear viscosity with concentration, which arises from the fact that chains in solution start overlapping (Figure 4A).^72^ Our findings agree well with those of Georgiades et al. who observed for MUC5AC mucin solutions purified from porcine stomach and porcine duodenum mucin solution a power law dependence of the shear modulus on concentration with an exponent of ∼2. The dependence of the effective shear viscosity on the mucin concentration in this study shows two sections: up to a concentration of 30 mg/mL the viscosity increases only slightly (exponent = 0.5) and from higher concentrations onward with an approximately 10-fold higher exponent. The study identified a critical concentration at which the mucin solutions transition from behaving like a Newtonian liquid to forming a viscoelastic polymer network.^36^ Similar scaling laws were also observed for mucus mixtures based on poly(acrylic acid) (PAA) mimicking porcine intestinal mucus (PIM).^65^

#### 3.3.2 Effect of Additives

After the establishment of the concentration dependence of the viscoelastic properties of BSM, we investigated how they become modified by the presence of different additives. To do so, we added different salts, proteins, or DNA to the mucus samples; all components that may be present to a larger or lesser extent in biological mucin samples and that are relevant for its function.

##### 3.3.2.1 Sodium

In that context, we examined the impact of sodium, as sodium ions could potentially alter the hydration state of mucus and thus may cause a change in its viscoelastic properties.^73,74^ For that purpose, we performed rheological measurements with 100 mg/mL BSM solutions with four different sodium concentrations, where one may expect the main effect arising from an increase in the ionic strength of the solution and only a lesser effect from specific binding of the ions to the mucins. The resulting BSM solutions had sodium concentrations of 0.19, 0.24, 0.38, 2.19, and 5.18 M. A 100 mg/mL BSM solution dissolved in DPBS contains already 0.19 M sodium, resulting from manufacturing of the commercial BSM. The pH values of all solutions were around 6.9.

Previous experiments on mucins purified from tracheobronchial mucus secretions from CF patients and healthy subjects suggested a less extended flexible coil structure in the presence of sodium.^75^ The authors aimed to investigate the impact of sodium concentration on mucin aggregation and the resulting viscoelastic properties of CF lung mucus secretions, which are essential for effective mucus clearance due to their transport capacity. Structural changes in the internal architecture of the mucins due to the addition of sodium may also occur in sodium-containing BSM solutions, which then should also affect the rheological behavior. It seems that a small increase in salinity can enhance the mucus clearance from the respiratory tract (tested here up to 90 mM),^76^ which should be related to the reduction of the viscoelastic properties.

According to the rheological measurements shown in Figures 4B and S6, the addition of sodium to a 100 mg/mL BSM solution led to a generically similar increase in the viscoelastic moduli *G*′ and *G*″ with increasing frequency for all five sodium concentrations. The absolute values first decreased with the concentration of added sodium but then increased again at very high sodium concentrations (Figure S6A). Here, the solution with 0.24 M sodium in a 100 mg/mL BSM solution had lower values of *G*′ and *G*″ value at low frequencies by almost a factor 5 compared to the solution with 0.19 M sodium, which was an interesting effect for the addition of just 50 mM sodium.

The phase angle (Figure S6B) shows that the relative relevance of the elastic component is first reduced with increasing sodium concentration and then became more significant again for the two highest sodium concentrations. However, here it has to be noted that in these samples with 2.19 and 5.18 M sodium almost all of the water molecules are directly bound to the ions and therefore one may effectively see a more concentrated BSM network. In addition, for most viscoelastic polymer solutions it is observed that with increasing frequency the relative contribution of the elastic component becomes more important,^77^ but for the BSM solutions an opposite tendency was noted. This is an intriguing behavior that shows that for higher frequencies, the ability of the BSM hydrogel to dissipate energy increases compared to its ability to store energy elastically. These findings suggest that the primary effect arises from an increase in the ionic strength rather than specific ion binding to mucins.

For all samples, shear thinning behavior was observed in the steady shear viscosity experiments (Figure S6C). The 0.24, 0.38, and 2.19 M NaCl solutions showed similar curves, starting from just under 1000 mPa·s for low shear rates and then decreasing to 100 mPa·s at higher shear rates. In contrast, the highest sodium concentration of 5.18 M showed a shear viscosity value of higher than 1000 mPa·s at low shear rates, decreasing more rapidly with increasing shear rate and reaching the same values as the other sodium BSM solutions at a shear rate of around 1.0 s^–1^ (Figure 4B).

##### 3.3.2.2 Calcium

To investigate the effect of calcium ions (CaCl_2_), which is known to have an important influence on mucus network properties,^1,9,21^ we studied 100 mg/mL BSM solutions with different calcium concentrations. The 100 mg/mL BSM solution contained already an initial concentration of 0.1458 mg/mL calcium (=3.75 mM) (see the SI, ICP-OES) and it was dissolved in a HEPES buffer that contained no additional calcium. The final calcium concentrations of the solutions were 3.8, 5.3, 8.8, 14.0, 24.2, 51.4, 61.6, 78.6, 100.4, and 150.0 mM Ca^2+^. The pH value of all solutions was around 6.7.

The addition of calcium led to substantial changes of the rheological properties already at rather low Ca^2+^ concentrations (Figure 4C), different from the situation observed for the addition of sodium (Figure 4B). A continuous increase of the viscoelastic moduli was seen with increasing calcium concentration, where the maximum was reached for the 51 mM samples (the power law exponent for the dependence on the Ca^2+^ concentration is 0.6). However, a further increase to 62 mM Ca^2+^ led to a substantial reduction, and for the sample with the calcium concentration of approximately 79 mM, a concentration far above that found in nature, the moduli became reduced again by about a factor 10 and were lower than the moduli of the solution without added calcium. These low moduli remained upon increasing the Ca^2+^ concentrations further to 100 and 150 mM (Figures 4C and S7A,B). This reduction of the viscoelastic properties is likely related to the compaction of mucins (e.g., MUC5B) that has been reported in the presence of excess Ca^2+^ ions.^21,78^ We detected several crossover points for these low concentrations, which are better seen in the phase angle depicted in Figure S7C. When performing frequency sweeps up and down, we noted identical curves, indicating reversible deformation of the samples.

For the phase angle (Figure S7C), rather constant values were observed with a slight increase with increasing frequency. The sample with 51 mM calcium not only had the highest moduli but also the elastic part was most pronounced. The samples with low calcium concentration (3.8 and 5.3 mM) showed *G*′ and *G*″ values around 0.5 Pa increasing up to 5 Pa with increasing frequency, i.e., somewhat more marked viscous properties (phase angle increased to 65° at higher frequencies).

The steady shear viscosity measurements with the same calcium BSM solutions showed a similar shear thinning behavior as for the pure BSM solutions, but at low shear rates, the viscosity was significantly higher compared to the pure BSM solution (Figure S7D). At a shear rate of 1.0 s^–1^, we observed the highest viscosities for 24 and 51 mM Ca^2+^, being higher by a factor of 100 (Figure 4C). However, they drastically decreased with increasing shear rate; reaching at a shear rate of around 1000 s^–1^ the same values of about 100 mPa·s, irrespective of calcium concentration. This means the shear thinning effect was much more pronounced for the samples with 24 and 51 mM calcium, and these samples were also more resistant with their viscous properties against shear forces. The samples with more added calcium (79, 100, and 150 mM) showed a much less pronounced decrease in viscosity with increasing shear rate, with a lower slope, leading to viscosities in the same range as for the low (5.3 mM) or no (3.8 mM) added calcium.

Apparently, BSM showed a rather marked sensitivity to the presence of Ca^2+^. This can be explained by the fact that the 100 mg/mL BSM solution was experimentally determined to have a total protein content of approximately 85 mg/mL (refer to the Methods section), with around 10% of it being sialic acid in free and glycosidically bound form within the mucins. This estimate is based on experimental data from previous studies^79,80^ and corresponds to about 8.5 mg/mL or 25–30 mM of sialic acid. At this sialic acid concentration, marked cross-linking effects are anticipated with the addition of 10–20 mM calcium, as was observed in the experiments. The calcium added to the BSM solution is likely to bind to the carboxylic groups of the sialic acid, along with other acidic groups in the mucin backbone or calcium-binding sites, thereby leading to a cross-linking of chains. At very high calcium concentrations, the excess of Ca^2+^ should lead to oversaturation of the carboxylate groups and thereby to reduced cross-linking. This aligns with the observation that above ∼79 mM Ca^2+^, we observed a noticeable decrease in the viscoelastic properties.

In summary, it can be stated that the addition of concentrations of about 10 to 50 mM calcium enhances the gel properties of the BSM system, where this effect was rather sensitive in its extent to the calcium concentration.

The sensitivity of mucus systems to calcium has been observed in various solutions such as PGM and MUC5B solutions purified from human saliva. An increase in viscoelastic parameters was found with the addition of calcium concentrations of approximately 10 mM. This increase was ascribed to stronger entanglement of the mucins and the resulting more compact network structure.^9,21^ For example, in CF the deficiency of CFTR-mediated bicarbonate transport results in more free calcium, which can contribute to mucin complexation.^78^

##### 3.3.2.3 Magnesium

For comparison, we also studied the influence of magnesium ions (from MgCl_2_) as another biologically relevant divalent cation on the BSM rheology. The behavior of magnesium and mucus has been less studied than that of calcium, but it is assumed that it behaves similarly with regard to viscoelastic properties.^3^ The magnesium concentrations, which were added to 100 mg/mL BSM solutions, were similar to those previously investigated for calcium in order to ensure comparability. The initial concentration of magnesium in a 100 mg/mL BSM solution was 1.40 mM (SI, ICP-OES), and it was dissolved in a HEPES buffer that contained no additional magnesium. The final Mg^2+^ concentrations of the solutions were 1.4, 5.0, 8.8, 13.4, 24.0, 49.4, 76.4, 101.4, and 151.4 mM Mg^2+^. The pH values of all solutions were approximately 6.7.

The addition of magnesium resulted in substantial alterations to the rheological properties, even at low concentrations (Figures 4D and S8) that were similar to those observed with calcium. With an increasing magnesium concentration, an increase in the viscoelastic moduli was observed (power law exponent = 0.9) up to a concentration of 13.4 mM, at which a maximum was reached. At concentrations of up to 24 or 50 mM, the moduli then decreased again to a level comparable to that of a BSM solution without added magnesium but not lower. This level was maintained even at higher magnesium concentrations (101 and 151 mM) (Figure S8A,B). This behavior corresponds to the observations made with the addition of calcium, with the exception that the maximum of the viscoelastic moduli for magnesium was already observed at approximately 14 mM, in contrast to 51 mM for calcium (Figure 4C,D). In addition, the values of the viscoelastic moduli did not decrease as strongly as for calcium, the lowest values for magnesium were about a decade higher than the lowest values of calcium observed for the three highest ion concentrations (∼75, 100, and 150 mM). From a mechanistic perspective, processes analogous to those described above for calcium may occur, i.e., binding to the negatively charged acid groups of sialic acid or other acids, which then might result in compaction or elongation of mucins. It is noteworthy that the enhancement of the viscoelastic properties at lower concentrations was more pronounced for Mg^2+^ than for Ca^2+^.

The phase angle (Figure S8C) indicated that at the lower concentrations of magnesium (5.0 and 8.8 mM), the elastic component in the system was dominant, while at the intermediate and high magnesium concentrations, the viscous component was more pronounced. In general, and independent of concentration, the viscous component increased with increasing frequency.

Shear thinning behavior was observed in the steady shear viscosity tests (Figure S8D). The highest values (approximately 4000 mPa·s) at a shear rate of 1.0 s^–1^ (Figure 4D) were found for the BSM solution with 13.4 mM magnesium, which also exhibited the highest viscoelastic moduli. As the shear rate increased, the viscosities of all solutions decreased to a common value of approximately 100 mPa·s, a phenomenon also observed for calcium.

##### 3.3.2.4 Lysozyme

Apart from small ions, native mucus is surrounded by a variety of nonmucin proteins; hence, their contribution to the viscoelastic characteristics of mucus needs to be understood. For this purpose, we studied the effect of lysozyme acting here as a model protein on the viscoelastic response of BSM.

Lysozyme, a key component in various biological fluids, plays a crucial role in mucosal defense due to its antimicrobial properties, achieved through the hydrolysis of β-(1,4)-glycosidic bonds in the peptidoglycan of pathogenic microorganisms’ cell walls,^81^ and its anti-inflammatory effects.^82^ Its interaction with mucins, such as the ability to form complexes with porcine gastric mucin (PGM) through various binding types,^81^ underscores its significance in mucosal biology and prompted our investigation into its effects on BSM. It is worth noting that the BSM did not inhibit lysozyme activity, which contrasts with the PGM that did so under certain conditions.^83^

Here, 100 mg/mL BSM solutions with 1.0, 2.0, or 10.0 mg/mL lysozyme were measured at a pH value of 7.2. At this pH, lysozyme with an isoelectric point of 11 is fully positively charged (∼8 positive charges) and may act as a cross-linking agent via electrostatic interactions between negatively charged mucins and positively charged lysozyme.^81^

The data given in Figure 4E show that for the lower lysozyme concentrations, the rheological moduli did not change much compared to the situation without added lysozyme, and the loss modulus *G*″ was larger than the storage modulus *G*′ within the investigated frequency range, resulting in a constant phase angle at 70° for both lysozyme concentrations (Figure S9A,B). For 10.0 mg/mL lysozyme the moduli were much larger (Figure 4E) and here *G*′ was about the same as *G*″ (i.e., a much lower phase angle δ (Figure S9B)). Interestingly, this sample differed even more with respect to the viscosity, as it had a much higher viscosity, which was also visible by the eye. The viscosity decreased to the level observed in low lysozyme concentration solutions at high shear rates (100 mPa·s) with a rather strong power law with an exponent of 0.93 (Figure S9C). The much more marked viscosity of the BSM sample with 10.0 mg/mL lysozyme added was not due to the relatively high concentration of lysozyme, as shown by a comparative measurement of a sample with the same lysozyme concentration but without BSM, which showed a 100–1000 times lower viscosity (Figure S9C). Therefore, this behavior is likely due to electrostatic interactions between the positively charged groups on the lysozyme and the negatively charged sites on the mucin molecules.^81,84^ This observation was also noted in sputum from patients with chronic obstructive pulmonary disease (COPD) to which lysozyme was added.^84^

##### 3.3.2.5 DNA

Finally, we investigated the effect of DNA on mucus viscoelastic properties, which are known to be influenced by DNA^39^ e.g., in the context of neutrophilic granulocyte activation, which burst and release DNA during inflammation. Especially, in chronic muco-obstructive lung diseases such as cystic fibrosis,^85,86^ therapeutic strategies are based on DNase-supported mucin dilution.^39^ We studied 100 mg/mL BSM solutions with varying concentrations of DNA. The determination of the initial DNA content in a 100 mg/mL BSM solution showed 1.2124 mg/mL DNA with a standard deviation of ±0.000743 mg/mL. In small steps of 5 mg/mL, we increased the DNA concentration up to 20 mg/mL. The pH value of the solutions was around 7.07.

The solution with the smallest amount of DNA (baseline 1.21 mg/mL) had the highest values for the viscoelastic moduli, with *G*″ being markedly higher than *G*′. The DNA concentration of 15 mg/mL came close to these values, but with an increasing DNA concentration, the viscoelastic moduli decreased, and there were nearly no differences between the *G*′ and *G*″ (Figures 4F and S10A,B). All of the solutions showed shear thinning behavior with nearly the same power law exponent of around 0.3 (Figure S10C). Again, the 15 mg/mL concentrated DNA solution had the highest viscosity value but approached very quickly, already at 1 s^–1^ shear rate, the values of the other solutions with added DNA.

Initially, it was expected that the viscoelastic moduli would increase with increasing DNA concentration in the BSM. Interestingly, as the DNA concentration increased, the viscoelastic moduli decreased and the difference between *G*′ and *G*″ narrowed, suggesting that higher DNA concentrations alter the viscoelastic balance in BSM solutions. It may be hypothesized that the DNA concentrations added were already too high to be rheologically visible with the mucus structure. This trend, along with the observation of consistent shear thinning behavior across different DNA concentrations, highlights the complex interaction between DNA and mucin molecules, underscoring the importance of DNA concentration in modulating the rheological properties of mucus-like systems.

Lehr et al. pointed out the importance of DNA in mucus models.^10^ They compared the rheological behavior of various mucus surrogates with that of human native pulmonary mucus, taking into account the elastic-dominant behavior when selecting components for the mucus model. This partially simulates the rheological behavior of native pulmonary mucus. To approximate the viscoelastic properties of human airway mucus with the BSM, our study suggests making specific adjustments, such as adapting the elastic and viscous fractions within certain limits using the described calcium and DNA concentrations, which would enable predictions to be made using the FKVM.

### 3.4 Detailed Analysis of the Rheological Data

**Figure 5:**
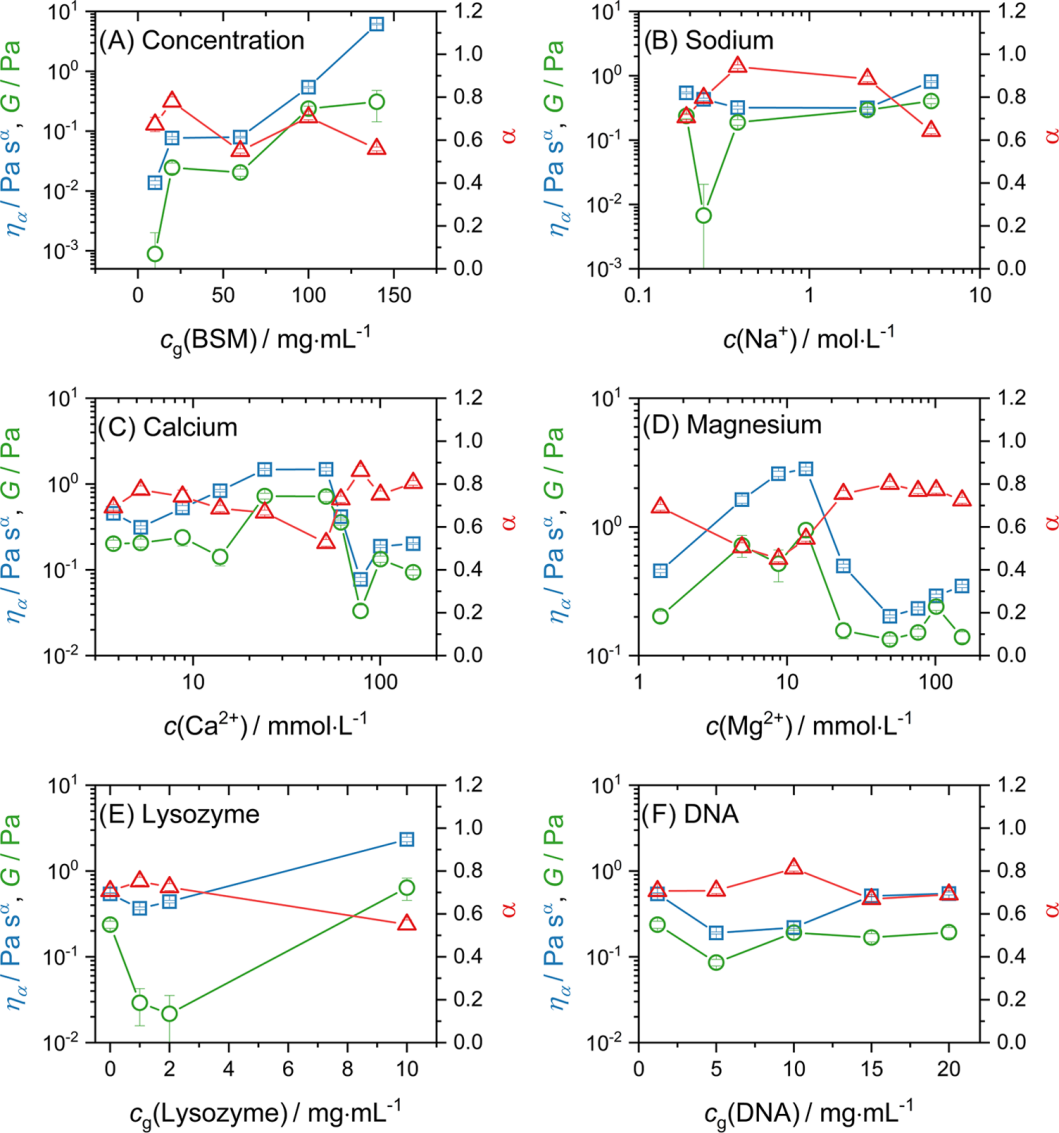
FKVM fit parameter results for (A) the BSM concentration series, (B) sodium, (C) calcium, (D) magnesium, (E) Lysozyme, and (F) DNA.

The frequency-dependent rheological data clearly demonstrated an interesting viscoelastic behavior of the BSM hydrogels, which must result from a wide spectrum of relaxation times (i.e., cannot be described by a single relaxation time as for instance given by the Maxwell model). In order to gain further insights into the viscoelastic properties, we analyzed the different data sets with a fractional Kelvin–Voigt model (FKVM), which should be suited to describe our data as it applies to materials that are dominated by their elastic properties on longer time scales, as observed in our experimental data. The fit parameter results for the concentration series and the additives are shown in Figure 5. All fits are shown in the Supporting Information.

As a function of the overall BSM concentration (Figure 5A), the fractional exponent α was rather constant at a value of around α = 0.70, although there was a small decrease toward high concentration. This indicated that, in general, BSM solutions are well described by a model consisting of one purely elastic spring and one relatively viscous spring-pot (α ≈ 0.6–0.7) in parallel. For both η_α_ and *G*, we first saw a steep increase from 10 to 20 mg/mL, followed by rather constant values until 100 mg/mL. In this rather wide concentration regime, surprisingly, the viscoelastic properties hardly changed, which is very different from what is normally observed in viscoelastic polymer solutions.^87^ This could be an important aspect for its *in vivo* performance, as apparently its general viscoelastic properties change only rather little over an extended concentration regime. Going to 140 mg/mL, η_α_ increased much more strongly than *G*, indicating that the relative importance of the viscous properties versus the elastic properties became larger.

As a function of the additive concentration, variation of the model parameters was generally rather small. Upon further examination, several interesting features merit attention.

For sodium, there was a slight increase of both η_α_ and *G* toward higher concentrations. Although the influence was rather small, Na^+^ thus tended to strengthen the network structure, most likely through electrostatic interaction with negatively charged parts of mucins or other proteins that can be found in BSM. This could result in changes to the structure of the mucin, as well as an effectively higher concentration of mucin, due to the increased hydration of the ions.^75^

The BSM solutions showed a much more pronounced dependence on added Ca^2+^ compared to the addition of Na^+^. With increasing calcium concentration, both η_α_ and *G* increased until a maximum was reached between 24 and 51 mM. When the concentration was increased further, a marked decrease of η_α_ and *G* was observed, which reflects the known compaction of MUC5B, already described above. The increase of η_α_ and *G* was mirrored by a decrease of α, which indicates that the BSM solutions’s response becomes significantly more elastic.

For magnesium (Figure 5D), a very similar trend was found. Both η_α_ and *G* increased until a maximum was reached at c(Mg^2+^) = 13.4 mM before decreasing again, while α showed the opposite trend. Both divalent cations seem to induce the same change in the mucin structure, which leads to a much more elastic response in a small concentration window. Interestingly, the maximum was shifted to lower concentrations for magnesium and also showed higher values.

A concentration dependence was noted for the addition of lysozyme, where *G* first decreased up to 2 mg/mL before increasing again for the highest concentration at 10 mg/mL. The elastic properties were first weakened and then strengthened by the presence of lysozyme. η_α_ showed only a slight decrease before significantly increasing at 10 mg/mL.

In contrast, the addition of DNA to a BSM solution did not seem to have any significant effect on the viscoelastic properties of the BSM.

Overall, the rheological properties of the BSM solution seem to be largely unaffected by the presence of sodium, lysozyme, and DNA. The behavior for addition of calcium and magnesium is very different, and one observes a very marked influence on the FKVM parameters at relatively low concentrations in the mM regime.

The FKVM allows a complete description of the complex viscoelastic behavior of mucus, with a low number of model parameters, which would be impossible with classical spring-dashpot models. This is mainly due to the wide spectrum of relaxation times inherent in BSM. The FKVM, particularly suited for materials exhibiting power law behavior and dominance of elastic properties at longer time scales, is an ideal choice for our analysis as it effectively describes materials with a broad range of microstructural length and time scales, typical of many biological systems including BSM.

### 3.5 Proteome

**Figure 6:**
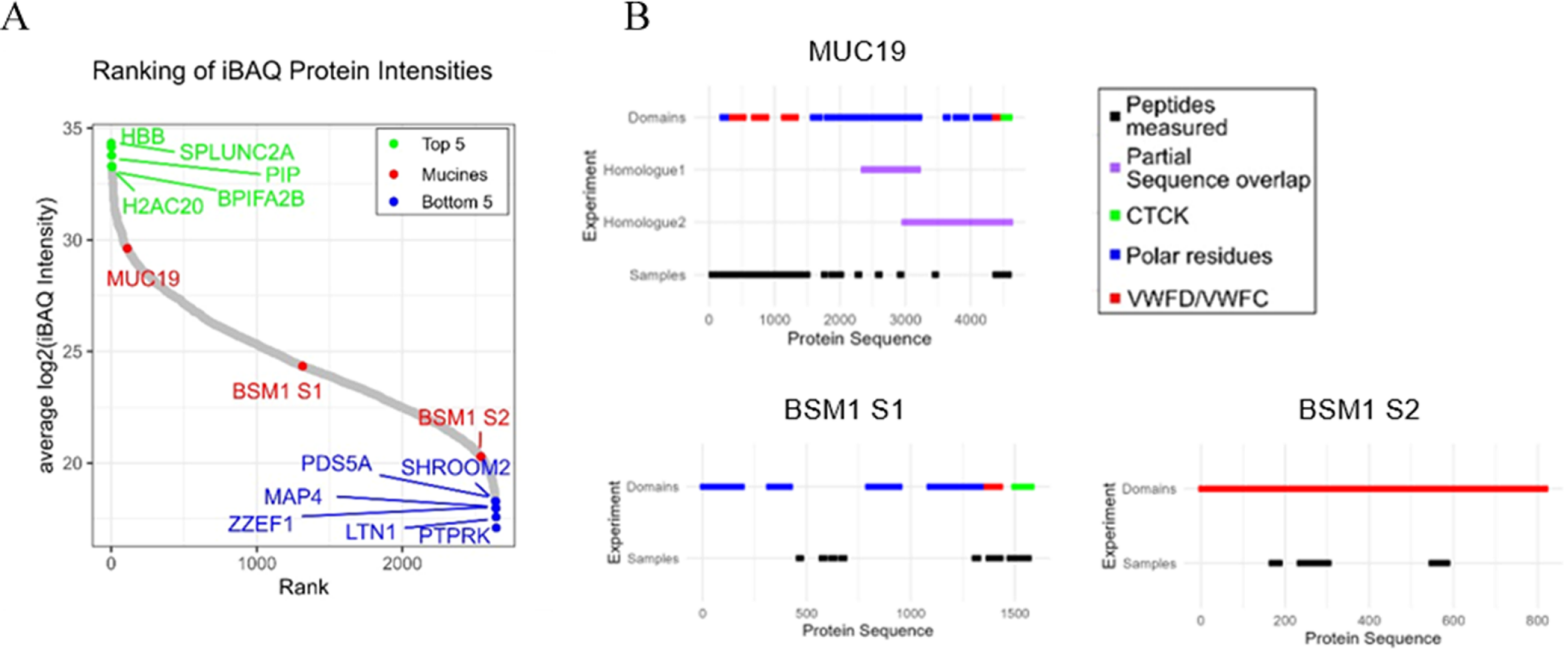
Ranking of quantified proteins according to their average log2 iBAQ normalized intensity. (A) Top 5 and bottom 5 proteins are highlighted in addition to the quantified mucins MUC19, BSM1 S1 (UniProt ID Q9N1P0), and BSM1 S2 (UniProt ID O62672). (B) Peptides measured in the three technical replicates for each mucin are highlighted together with overlapping sequences (purple), and the three different domains: carboxyl-terminal cystine knot (CTCK) (green), von Willebrand Factor type D and C (VWFD/VWFC) domains, and the polar residue-rich domain (proline, theronine, and sering (PTS)) as annotated on UniProt.

Commercial mucins underwent several purification steps before being sold as lyophilized powder. We surmised several other proteins and DNA inside of commercial BSM and therefore conducted proteomics and DNA content analysis. Overall, we identified and quantified 2645 proteins in the replicates (Figure 6A). Three of these 2645 proteins were mucins: MUC19, and two different submaxillary mucin fragments that we numbered with sequence 1 (S1, UniProt ID Q9N1P0) and sequence 2 (S2, UniProt ID O62672) as they have the same gene symbol BSM1. All three proteins have partially overlapping sequences, but unique peptides were identified for each of them (Figure 6B). These mucins determined in this examination highlighted the diversity within the protein composition of BSM.

## 4 Conclusions

In conclusion, the comprehensive investigation of Bovine Submaxillary Mucin (BSM) in this study provides valuable insights into its biomechanical behavior in the context of several physiologically relevant stimuli and presents a multiparametric study of the viscoelastic responses of this biomaterial. Our findings reveal that the viscoelastic properties of BSM only somewhat increase with material concentration, which is important for robustness in biological systems. Addition of calcium and magnesium ions markedly enhances the viscoelasticity of BSM, while changes in ionic strength resulting from NaCl have minimal effects. The application of a mucus analysis strategy using the fractional Kelvin–Voigt model captures the complete rheological behavior of the BSM, utilizing a minimal set of model parameters. Cryo-scanning electron microscopy effectively revealed the intricate internal network structure of BSM, facilitating a detailed analysis of pore sizes, while complementary proteome analysis provided a deeper understanding of the BSM composition, which identified MUC19 as the predominant gel-forming mucin in the BSM.

Our findings and modifications to the BSM offer a solid foundation for developing targeted human airway mucus models, which can be adjusted to meet specific research requirements. Ribbeck et al. have previously discussed the challenges associated with this, in their research on mucus surrogates derived from tissue-specific mucins to simulate various types of human mucus.^16^ Although it is feasible under certain conditions to directly tailor the physicochemical properties of these models to match human mucus sources, it remains a complex task, particularly when considering the full spectrum of mucus characteristics across various length and time scales. However, the modifications to the BSM explored in our study provide a robust foundation for developing targeted human airway mucus models that can be fine-tuned to meet specific research requirements. This approach allows for more accurate simulations of human mucus behavior, enhancing our understanding of and potential treatment strategies for various respiratory conditions.

In comparison to other studies on mucins and mucus, the BSM rheological behavior BSM makes it an important mucus source to study responses to different physicochemical situations relevant for mucus dynamics under physiological and pathophysiological conditions. Our analytic FKVM provides researchers from various fields with a fundamental basis to study this biomaterial as a surrogate for human lung mucus in greater detail.

## Supporting Information Available

Additional experimental details, materials, and methods, as well as complementary data (PDF)
Protein sequence, leading razor protein, start and end positions, identification type, intensity, and LFQ intensity (XLS)
