# Supplementary material for: Comprehensive Characterization of the Viscoelastic Properties of Bovine Submaxillary Mucin (BSM) Hydrogels and the Effect of Additives
Source: Biomacromolecules. 2024 Jun 4;25(7):4014–29. doi: 10.1021/acs.biomac.4c00153 (PMC11238336; doi:10.1021/acs.biomac.4c00153)
Supplement: Supplementary file 1 — bm4c00153_si_001.pdf [file bm4c00153_si_001.pdf]

Supplementary Information

Comprehensive Characterization of the Viscoelastic Properties of Bovine Submaxillary Mucin (BSM) Hydrogels and the Effect of Additives

Hanna Rulff, Robert F. Schmidt, Ling-Fang Wei, Kerstin Fentker, Yannic Kerkhoff, Philipp Mertins, Marcus A. Mall, Daniel Lauster, Michael Gradzielski

1. Methods

1.1 Measurement of the calcium and sodium content in BSM with Inductively Coupled Plasma - Optical Emission Spectrometry (ICP-OES)

BSM solutions with concentrations of 1 mg/mL (five repetitions) and 5 mg/mL (five repetitions) were prepared using a solvent mixture of distilled water, nitric acid 65 % (Suprapur, CAS-number: 7697-37-2, Merck KgaA, Darmstadt, Germany) and hydrogen peroxide 30 % (Nr. 476.1011, ChemSolute, Th. Geyer GmbH & Co. KG, Renningen, Germany) in a ratio of 5:3:1. The solutions were stirred as stated in the *Methods* section. The solutions were digested by a Microwave digestions system (Discover SP-D, CEM GmbH, Kamp-Lintfort, Germany) with a fixed heating and cooling protocol; first heating up to 160 °C at 17 bars for 20 min and then cooling down to 70 °C at 17 bar for 10 min. Afterwards the solutions and reference solutions of four different concentrations 0.1, 1.0, 10.0, and 100.0 mg/L (Multielement Standard Solution 5 for ICP, 54704, Merck KGaA, Darmstadt, Germany) were analyzed for their sodium, calcium and magnesium content by using Optical Emission Spectrometry with Inductively Coupled Plasma (ICP-OES) with a Varian ICP-OES 715 ES spectrometer. The parameters of the measurement were the following: plasma gas 16.5 L/min (argon), assistance gas 1.5 L/min (argon), atomizing pressure 200 kPa, observation height 10 mm, stabilization time 25 s, time for sample intake 35 s, measurement time 10 s, pumping speed 15 upm, flushing time 60 s. The determination of the wavelength used for the evaluation of the data for sodium, calcium and magnesium was made based on the information in DIN EN 16943 - 2017-07 and DIN EN ISO 11885 - 2009-09; wavelength sodium 588.995 nm, calcium 317.933 nm and magnesium 279.077 nm.

1.2 Baseline sodium and calcium concentrations of BSM determined via ICP-OES

The sodium concentration in BSM solutions (100 mg/mL) of the two batches dissolved in water was determined by five independent measurements at a sodium-specific wavelength of 588.995 nm. The calculated sodium concentration is 1.1263 ± 0.0005 mg/mL.

In the same way, the calcium concentration was determined from five independent measurements at a wavelength of 317.933 nm. It is 0.1458 ± 0.0002 mg/mL for 100 mg/mL BSM solutions of the two batches (dissolved in water).

Accordingly, the magnesium concentration was determined from five independent measurements at a wavelength of 279,077 nm. It is 0.0245 ± 0.0005 mg/mL for 100 mg/mL BSM solutions of the two batches (dissolved in water).

1.3 Addition of sodium-, calcium- and magnesium chloride to a 100 mg/mL BSM solution

The production of the four BSM sodium solutions proceeded as follows: DPBS buffer, containing 8 mg/mL sodium chloride, was added to each 100 mg BSM (containing 1.12 mg/mL sodium [0.05 mol/L], which was determined from ICP-OES measurements) and each 3.00 mg, 11.00 mg, 117.0 mg and 292.0 mg sodium chloride (≥99.5 %, S9888, Sigma-Aldrich, Merck KgaA, Darmstadt, Germany). This resulted in four BSM solutions with the following sodium concentrations: 0.24 mol/L, 0.38 mol/L, 2.19 mol/L and 5.18 mol/L. A 100 mg/mL BSM solution dissolved in DPBS was taken as baseline reference having a 0.19 mol/L sodium concentration.

The production of the nine BSM calcium chloride solutions proceeded as follows: 20.0 mmol/L HEPES-buffer was added to 100 mg BSM (containing 0.15 mg/mL calcium [3.75 mmol/L], which was determined from ICP-OES measurements) and each 0.22 mg, 0.74 mg, 1.5 mg, 3.0 mg, 7.0 mg, 8.5 mg, 11.0 mg, 14.2 mg and 21.5 mg calcium chloride (via calcium chloride dihydrate, ≥99.0 %, C7902, Merck KGaA, Darmstadt, Germany). This resulted in nine BSM solutions with the following final calcium concentrations: 5.25 mmol/L, 8.78 mmol/L, 13.95 mmol/L, 24.16 mmol/L, 51.37 mmol/L, 61.57 mmol/L, 78.58 mmol/L, 100.35 mmol/L and 150.01 mmol/L. A 100 mg/mL BSM solution dissolved in HEPES-buffer was taken as baseline reference containing 3.75 mol/L calcium.

The production of the eight BSM magnesium chloride solutions proceeded the same way. 20.0 mmol/L HEPES-buffer was added to 100 mg BSM (containing 0.034 mg/mL magnesium [1.40 mmol/L], which was determined from ICP-OES measurements) and each 0.34 mg, 0.7 mg, 1.14 mg, 2.15 mg, 4.57 mg, 7.14 mg, 9.52 mg and 14.28 mg magnesium chloride (magnesium chloride anhydrous, ≥98.0 %, CAS-Nr.: 7786-30-3, Avantor, VWR International GmbH, Darmstadt, Germany). This resulted in eight BSM solutions with the following final magnesium concentrations: 4.97 mmol/L, 8.75 mmol/L, 13.40 mmol/L, 24.00 mmol/L, 49.40 mmol/L, 76.40 mmol/L, 101.40 mmol/L and 151.4 mmol/L. A 100 mg/mL BSM solution dissolved in HEPES-buffer was taken as baseline reference containing 1.40 mol/L magnesium.

1.4 Summary of the purification method of the BSM carried out by the manufacturer

The BSM used in our experiments was purchased from Merck KGaA, which utilizes this protocol originally described by Nisizawa and Pigman[1] for the preparation of the mucin. Below is a brief outline of the main steps:

1. Collection and Pre-treatment of Glands: Immediately after collection, the cattle submaxillary glands are frozen and kept until use. They are then thawed, cleaned of fat and connective tissue, and cut into small cubes (4-7 mm).
2. Extraction: The gland pieces are successively extracted with a phosphate buffer (0.01 M, pH 7.0) at 2-4°C for 24 hours each. The extracts are filtered, and the residual glands are squeezed to remove as much of the extract as possible.
3. Clot Formation and Collection: The extracts are centrifuged (20.000 rpm, 30 min), and the clear solution is adjusted to pH 3.5 with 0.05 M hydrochloric acid to induce clot formation. The clots are then removed by further centrifugation (1500 rpm, 15 min).
4. Dissolution and Neutralization: The clots are dissolved in a sodium hydroxide solution (pH 8-9), followed by dialysis against large volumes of water with mechanical stirring at 2-4°C for three days.
5. Lyophilization: After final centrifugation and filtration, the dialyzed solutions are lyophilized to obtain the mucin in a dry form.

The BSM from Merck, obtained in lyophilized powder form, has been prepared through these steps to ensure the preservation of its biochemical properties. This preparation method is critical for maintaining the functional integrity of the mucin, which is essential for the objectives of our study.

1.5 Quantitative analysis of Cryo-Scanning Electron Microscopy (cryo-SEM) images

The pore size distributions of the mucus samples were determined by automatic analysis of cryo-SEM images with a self-written Fiji [2] macro. The macro can be used to analyze single images and image stacks. First an image or stack must be opened in Fiji and then a region of interest (ROI) must be selected (e.g., whole image excluding scale bar). The macro can then be executed by pressing *Run*.

The ROI will be duplicated and renamed "crop" and a Gaussian filter with a pixel radius of 1 will be applied to reduce noise [3]. The images are then binarized by an Otsu-based threshold [4] to separate the pixels into two classes (pores, fibers) with minimized inter-class variance. The binary images named "MASK_crop" are then analyzed (Area, Feret) with the Particle Analyzer included in Fiji. The analyzed pores are outlined where pores touching the edges of the images are not included into the analysis to avoid a bias origin from incomplete pores.

The outlines of the detected pores are overlayed in red onto the original images ("Composite") for manual inspection of the detection quality. When the macro is finished, the original images

are retained, as well as the binary images, which can be saved for further analysis (e.g., with exclusion of small objects). Two data tables are created. The *Results* table contains the values of all single pores. The *Summary* table contains averaged values of all pores per image for quick comparison of different images. The values in the data tables are in pixel units and must be multiplicated with the respective pixel size (e.g., in nm) to get the correct pore size distributions. As a key marker for the pore diameter the minimum Feret is recommended [5] as it gives the best estimation of the diameter of a 3-dimensional object (pore) from a 2-dimensional projection (image). To reduce the influence of pixel noise artefacts, the minimal Feret diameter of the smallest perceivable pores was measured manually and determined to be at 10 nm. Therefore, only data points with a minimal Feret diameter of 10 nm or larger were included in further analysis.

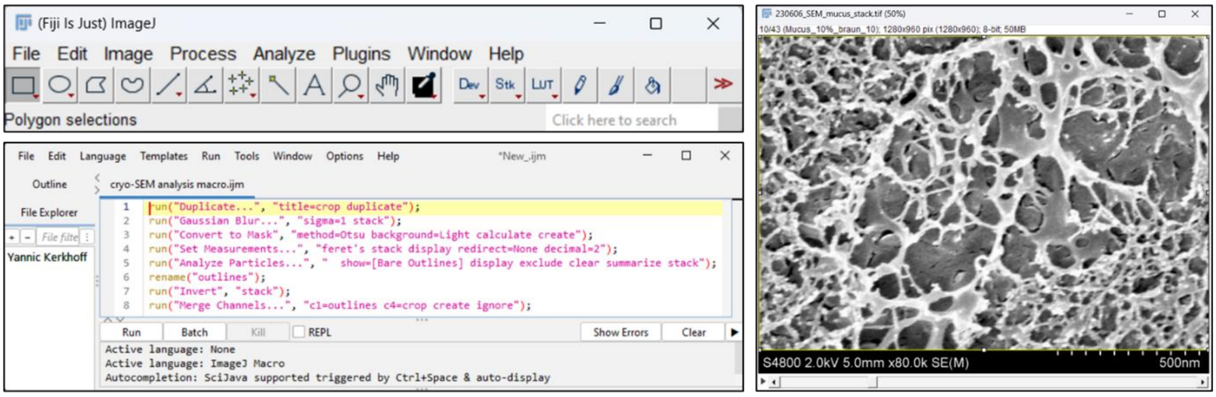

Figure S1A: *The Fiji action bar with the opened analysis macro (left) and the image stack containing cryo-SEM images with a selected ROI (yellow box) excluding the scale bar at the bottom.*

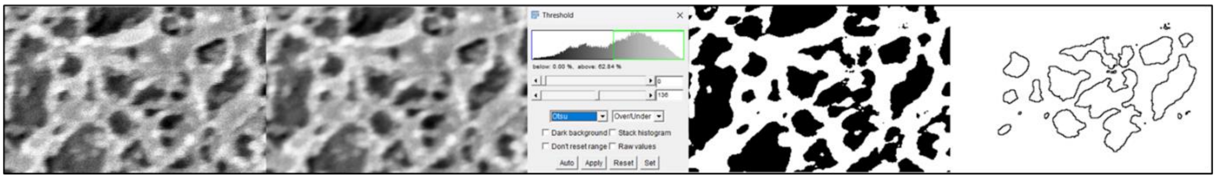

Figure S1B: *Representation of the basic image processing and analysis steps. From left to right: Crop of one original cryo-SEM image of Batch-Nr. LOT 3776068. A Gaussian filter with a pixel radius of 1 is applied to reduce noise. An Otsu-based threshold transforms the image into a binary image, with black pores and white fibers. The pores not touching the edges are detected and outlined by the Particle Analyzer.*

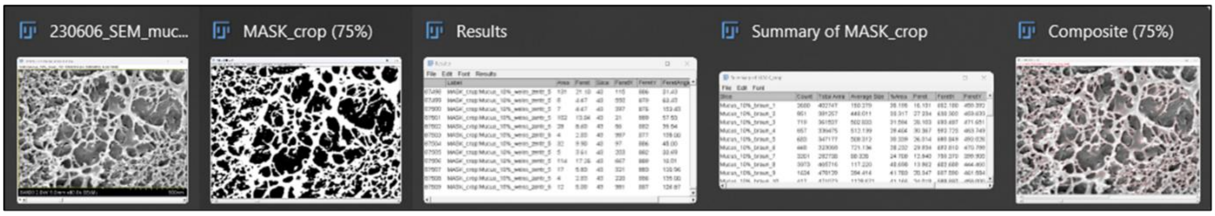

Figure S1C: *Output of the automated image analysis. From left to right: Original image (stack), binary result, Results table, Summary table, pore outline overlay for manual inspection.*

2. Results

2.1 Additional cryo-SEM images

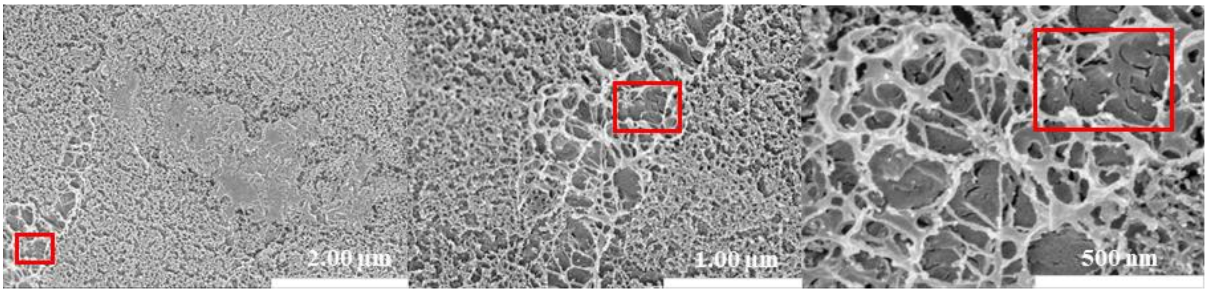

Figure S2: *Cryogenic-scanning electron micrographs (cryo-SEM) at 20.000 ×, 40.000 × and 80.000 × magnification of a 100 mg/mL BSM solution, Batch-Nr. LOT 3776068. Higher resolution structures are outlined in red.*

As a supplement to Figure 2 A and B, additional SEM images are shown here, also of a 100 mg/mL BSM solution of batch no. LOT 3829388. The lowest magnification showed mainly fine-meshed areas, as can also be seen in Fig 2A with batch no. LOT 3776068. Of course, there were also the larger meshed areas, as circled in red in the three ascending magnifications. Pore sizes of up to 500 nm are visible in this area. The violin plots (Fig 2C) clearly show the range of fine- and larger-mesh areas; the proportional distribution of small and large pores is also apparent.

2.2 Determination of the linear viscoelastic (LVE) region and the critical deformation

A B C

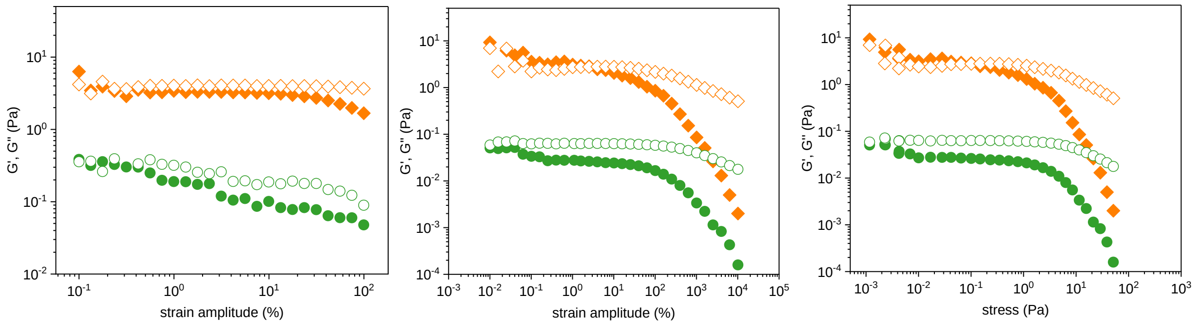

Figure S3: *Storage modulus G' (Pa) (filled symbols) and loss modulus G'' (Pa) (open symbols) as function of strain amplitude measured at 25 °C for BSM solutions of 20 (green circles) and 100 (orange rhombs) mg/mL (frequency: 1 Hz); (A) with a strain amplitude from 0.1 to 100 %, (B) 0.01 to 10000% and (C) plotted against the stress (Pa).*

The amplitude sweeps were performed at a constant frequency of 1 Hz. The linear viscoelastic (LVE) region was determined within the amplitude sweep. The LVE region represents the quasi-static behavior of the material commonly at lower strains and describes the range in which reproducible oscillatory measurements can be performed without structural deformation of the viscoelastic material[6, 7]. With an increase of the strain amplitude up to 100 % (Figure S3A) the values for G’ and G’’ of the 100 mg/mL BSM solution remained at constant around 4 Pa for the loss modulus that is dominating over the storage modulus (G’ around 3 Pa). The situation with a 20 mg/mL BSM solution is somewhat different, because here the values for G' and G" did not behave so constantly over the measured amplitude range but decreased slightly with increasing amplitude. However, G" remained the dominant modulus (G'=0.15 Pa and G"=0.3 Pa at 0.1 % strain). The values of the moduli were less stable overall and have more dispersion compared to the 100 mg/mL solution, which was due to the lower concentration of the BSM solution. Based on these measurement results, the LVE region was determined and the amplitude, which was then used for the subsequent frequency sweeps, was set to 1 %.

Furthermore, additional amplitude sweeps were performed with 20 mg/mL and 100 mg/mL BSM solution, where the amplitude was increased to higher values up to 10000% (Figure S3B). These showed a decrease for the storage modulus G' from an amplitude of 100 %. This value represents the critical deformation at which the mucus network is irreversibly changed. This is not in the LVE region. The corresponding yield stress value was around 0.7 Pa (Figure S3C). The decrease of the loss modulus occurs with a lower slope and can be observed at a deformation between 100 and 1000 % while G" remains dominant over G’ (middle).

2.3 Concentration Dependence of the Viscoelastic properties of BSM solutions

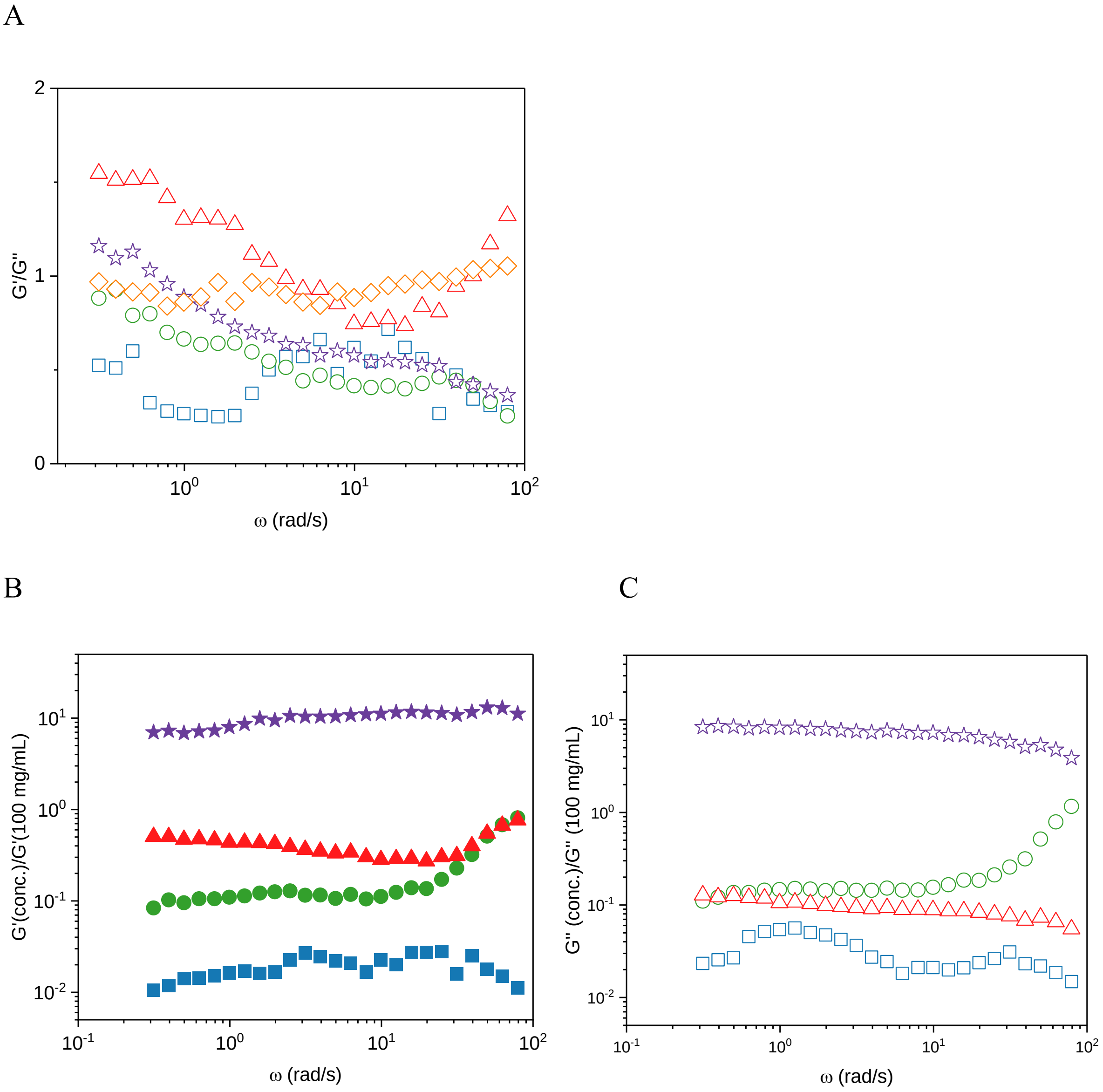

Figure S4: Viscoelastic moduli of different concentrated BSM solutions. *Storage modulus G' (Pa) (filled symbols) and loss modulus G'' (Pa) (open symbols) as function of angular frequency for BSM solutions of 10 (blue squares), 20 (green circles), 60 (red rectangles), 100 (orange rhombs), and 140 mg/mL (purple stars) (deformation: 1%). (A) Ratio of G'/G'' of each concentration as function of the angular frequency. (B) Ratio between storage modulus for a given concentration (10, 20, 60 and 140 mg/mL) and that obtained for 100 mg/mL as a function of the angular frequency. (C) Ratio between loss modulus for a given concentration (10, 20, 60 and 140 mg/mL) and that obtained for 100 mg/mL as a function of the angular frequency. Data shown are mean values of n=3 measurements for each concentration; the individual values are shown in Table S1.*

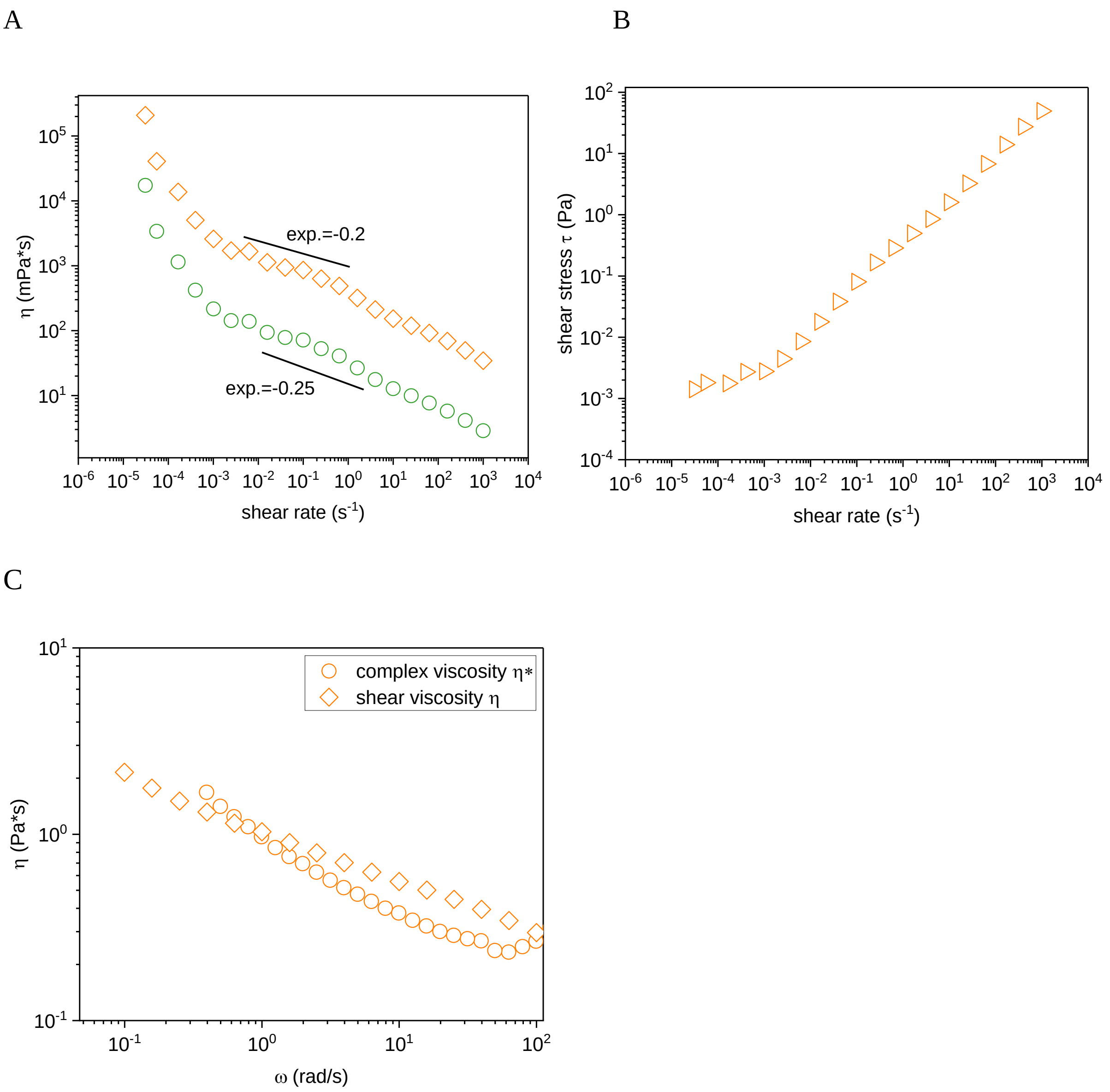

Figure S5: Steady shear viscosity of different concentrated BSM solutions*. (A) Steady shear viscosity as a function of shear rate, extended to very low shear rates for BSM solutions of 20 and 100 mg/mL. (B) Shear stress τ (Pa) as a function of shear rate (s^{-1}) for a 100 mg/mL BSM solution. (C) Comparison of the complex viscosity (Pa·s) and the shear viscosity (Pa·s) as a function of the angular frequency (rad/s) for a 100 mg/mL BSM solution displaying the Cox-Merz relationship, which is valid here. Data shown are mean values of n=3 measurements at the concentrations.*

When performing the viscosity measurement at low shear rates (0.00001 s^{-1}), an increase in the viscosity of a 20 mg/mL and 100 mg/mL BSM solution was observed. At the lowest shear rate of 0.00001 s^{-1}, the viscosity of the 20 mg/mL BSM solution was 20000 mPa·s (20 Pa·s) and the 100 mg/mL BSM solution is 200000 mPa·s (200 Pa·s) (Figure S5A).

Additionally, the shear stress (Pa) was plotted as a function of the shear rate (s^{-1}) (Figure S5B). By extrapolating the measured data from the yield curve to the point where it intersects with the shear stress axis, the yield point can be approximately determined, with a shear stress value of 1 $x10^{-3}$ Pa.

Furthermore, Figure S5C illustrates the complex viscosity (η^*) and shear viscosity (η) of a 100 mg/mL BSM solution plotted against the angular frequency. It can be observed that the complex viscosity measured under oscillatory shear closely corresponds to the shear viscosity measured under steady shear. The close alignment of these datasets confirms that the Cox-Merz relationship is valid for the BSM solution. This suggests that the dynamic and steady shear behaviors are equivalent at comparable rates, indicating similar microstructural behavior under both testing conditions.

2.4 Effect of additives on the Viscoelastic properties of BSM solutions

2.4.1 Sodium (Na^+)

A B C

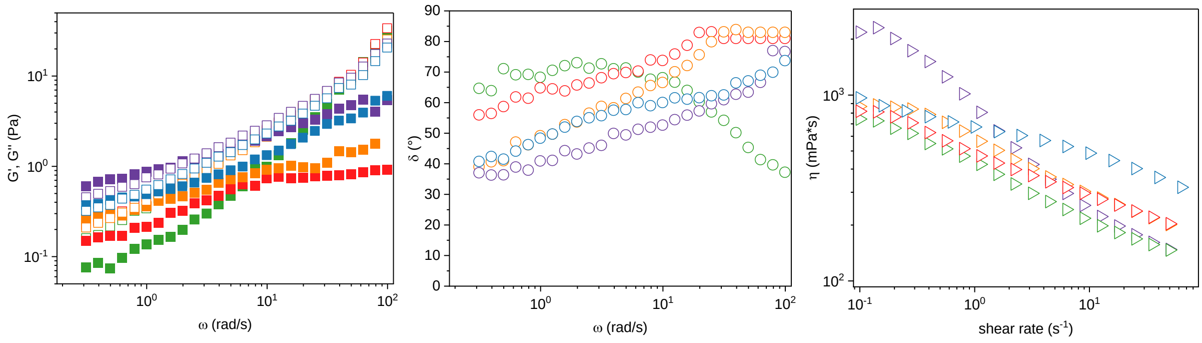

Figure S6: *(A) Viscoelastic properties of BSM solutions with added sodium (Na^+). Storage modulus G' (Pa) (filled squares) and loss modulus G'' (Pa) (open squares) and (B) corresponding phase angle δ (open circles) of 100 mg/mL BSM solutions with 0.19 M (baseline) (blue), 0.24 M (green), 0.38 M (red), 2.19 M (orange) and 5.18 M (purple) Na^+ were measured as function of angular frequency (rad/s) at 25 °C. (C) With a new aliquot of each solution the shear viscosity η (mPa·s) (open triangles) as a function of shear rate (s^{-1}) was measured. Data are shown as mean of n=3 measurements of each concentration. Individual values are shown in Table S1.*

2.4.2 Calcium (Ca^{2+})

A B

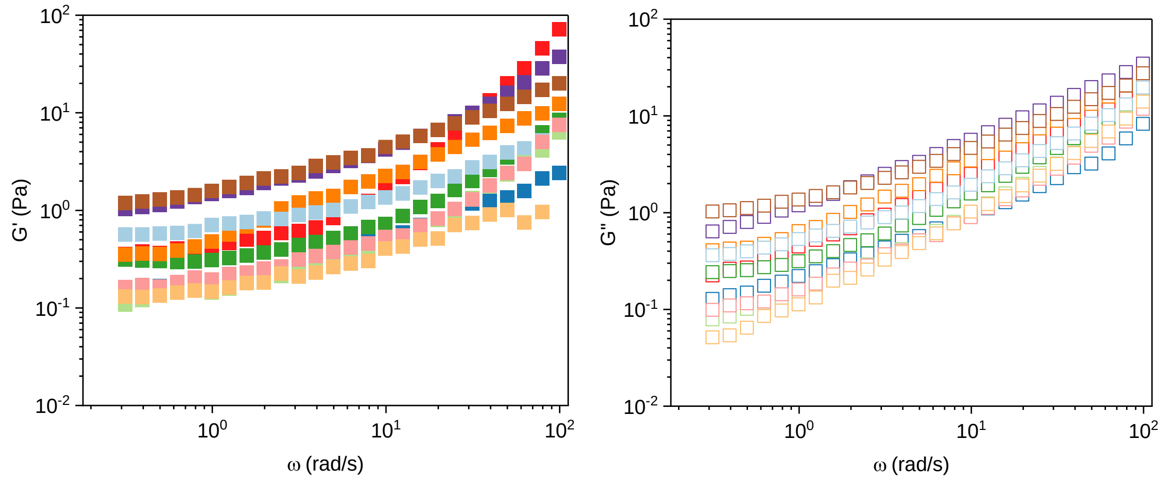

C D

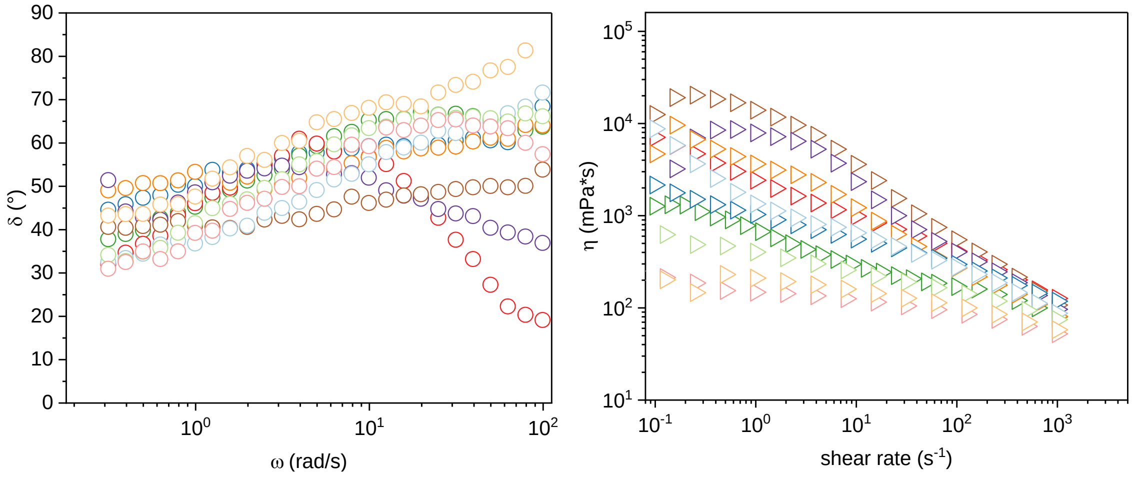

Figure S7: *Viscoelastic properties of BSM solutions with added calcium (Ca^{2+}). (A) Storage modulus G' (Pa) (filled squares) and (B) loss modulus G'' (Pa) (open squares) and (C) corresponding phase angle δ (open circles) of 100 mg/mL BSM solutions with 3.75 mM (baseline) (blue), 5.25 mM (green), 8.78 mM (red), 13.95 mM (orange), 24.16 mM (purple), 51.37 mM (brown), 61.57 mM (light blue), 78.58 mM (light green), 100.35 mM (light red) and 150.01 mM (light orange) Ca^{2+} were measured as function of angular frequency (rad/s) at 25.0 °C (D) With a new aliquot of each solution the steady shear viscosity η (mPa·s) as a function of shear rate (s^{-1}) was measured. Data are shown as mean of n=3 measurements of each concentration. Individual values are shown in Table S1.*

2.4.3 Magnesium (Mg^{2+})

A B

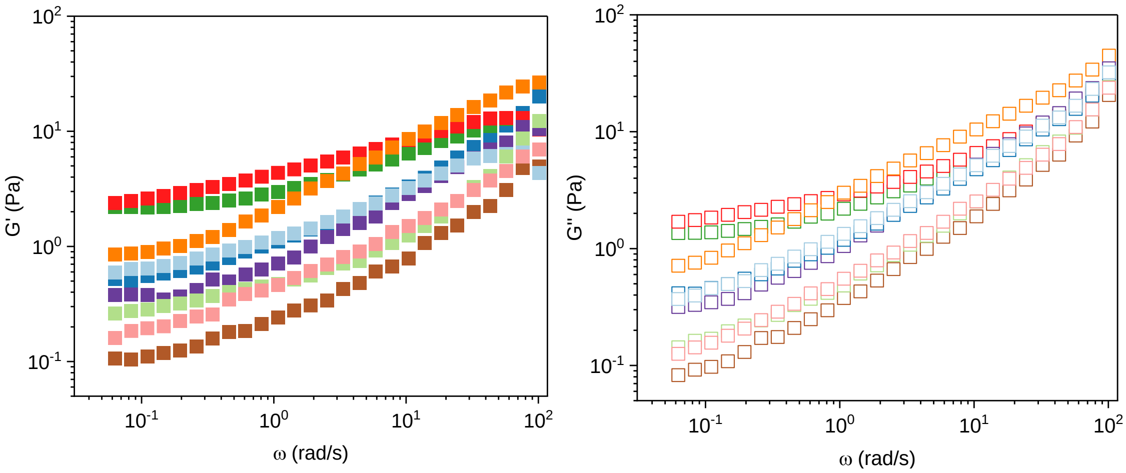

C D

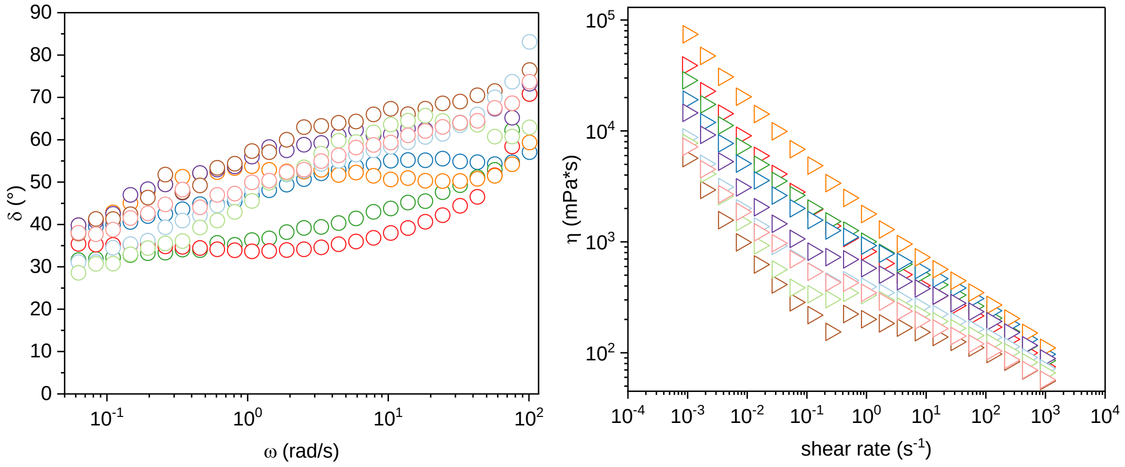

Figure S8: *Viscoelastic properties of BSM solutions with added magnesium (Mg^{2+}). (A) Storage modulus G' (Pa) (filled squares) and (B) loss modulus G'' (Pa) (open squares) and (C) corresponding phase angle δ (open circles) of 100 mg/mL BSM solutions with 1.40 mM (baseline) (blue), 4.97 mM (green), 8.75 mM (red), 13.40 mM (orange), 24.0 mM (purple), 49.4 mM (brown), 76.4 mM (light blue), 101.4 mM (light green) and 151.4 mM (light red) Mg^{2+} were measured as function of angular frequency (rad/s) at 25.0 °C (D) With a new aliquot of each solution the steady shear viscosity η (mPa·s) as a function of shear rate (s^{-1}) was measured. Data are shown as mean of n=3 measurements of each concentration. Individual values are shown in Table S1.*

2.4.4 Lysozyme

A B C

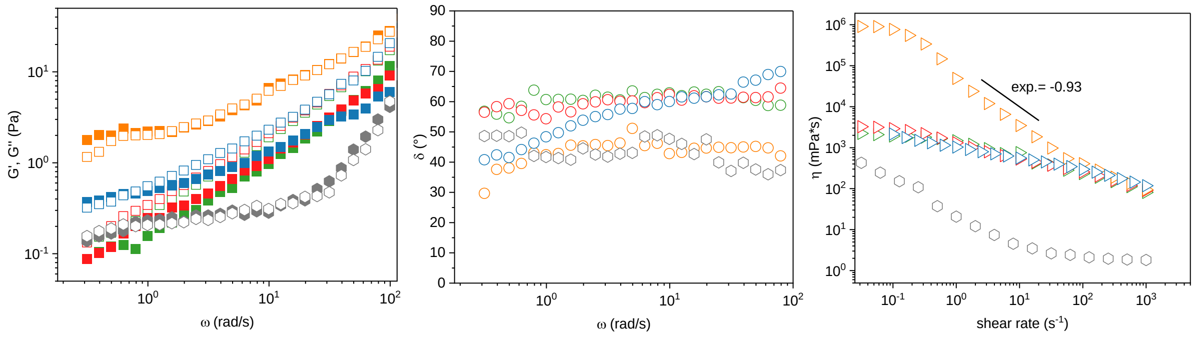

Figure S9: *(A) Viscoelastic properties of BSM solutions with added Lysozyme. Storage modulus G' (Pa) (filled squares) and loss modulus G'' (Pa) (open squares) and (B) corresponding phase angle δ (open circles) of 100 mg/mL BSM solutions with 0.0 (blue), 1.0 (green), 2.0 (red), and 10.0 mg/mL (orange) Lysozyme as well as a 10 mg/mL Lysozyme without BSM (grey symbol) were measured as function of angular frequency (rad/s) at 25.0 °C (C) With a new aliquot of each solution the shear viscosity η (mPa·s) (open triangles) as a function of*

shear rate (s^{-1}) was measured. Data are shown as mean of n=3 measurements of each concentration. Individual values are shown in Table S1.

2.4.5 DNA

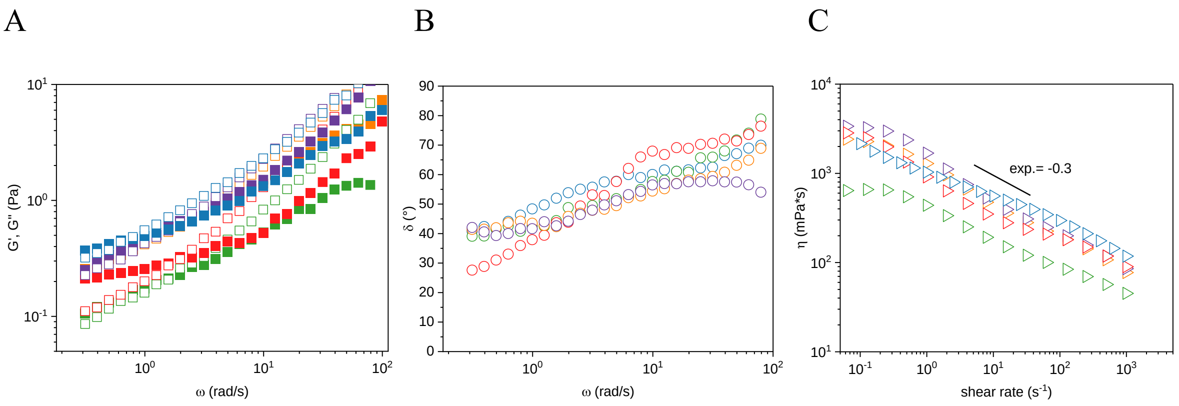

Figure S10: *(A) Viscoelastic properties of BSM solutions with added DNA. Storage modulus G' (Pa) (filled squares) and loss modulus G'' (Pa) (open squares) and (B) corresponding phase angle δ (open circles) of 100 mg/mL BSM solutions with 1.21 mg/mL (baseline) (blue), 5.0 mg/mL (green), 10.0 mg/mL (red), 15.0 mg/mL (orange) and 20 mg/mL (purple) DNA were measured as function of angular frequency (rad/s) at 25.0 °C (C) With a new aliquot of each solution the shear viscosity η (mPa·s) (open triangles) as a function of shear rate (s^{-1}) was measured. Data are shown as mean of n=3 measurements of each concentration. Individual values are shown in Table S1.*

2.5 Individual values of n=3 measurements, mean values and standard deviation

	Parameter	Individual values (n=3)	Mean value	Standard deviation
**Figure 1A**	G‘ (Pa) LOT 3776068	1.31/2.06/0.94	1.43	0.57
	G‘‘ (Pa) LOT 3776068	2.78/2.94/1.24	2.32	0.94
	G‘ (Pa) LOT 3829388	1.08/0.71/1.15	0.99	0.24
	G‘‘ (Pa) LOT 3829388	1.55/1.94/1.69	1.72	0.20
**Figure 2A**	G‘ (Pa) 10 mg/mL BSM	0.01/0.03/0.02	0.02	0.01
	G‘‘ (Pa) 10 mg/mL BSM	0.05/0.02/0.04	0.03	0.01
	η (mPa·s) 10 mg/mL	3.14/7.58/7.65	6.12	2.58
	G‘ (Pa) 20 mg/mL BSM	0.26/0.14/0.03	0.12	0.12
	G‘‘ (Pa) 20 mg/mL BSM	0.21/0.28/0.30	0.25	0.05
	η (mPa·s) 20 mg/mL	68.99/42.83/46.00	52.97	14.28
	G‘ (Pa) 60 mg/mL BSM	0.27/0.09/0.11	0.15	0.09
	G‘‘ (Pa) 60 mg/mL BSM	0.25/0.07/0.14	0.15	0.09
	η (mPa·s) 60 mg/mL	198/311/499	335	152
	G‘ (Pa) 100 mg/mL BSM	1.08/0.71/1.15	0.99	0.24
	G‘‘ (Pa) 100 mg/mL BSM	1.55/1.94/1.69	1.72	0.20
	η (mPa·s) 100 mg/mL	1062/970/1059	1031	52.27
	G‘ (Pa) 140 mg/mL BSM	9.40/11.60/11.10	10.72	1.15
	G‘‘ (Pa) 140 mg/mL BSM	12.40/14.30/11.60	12.71	1.39
	η (mPa·s) 140 mg/mL	7758/9937/8965	8892	1086
**Figure 2B**	G‘ (Pa) 0.19 M Na^+	1.08/0.71/1.15	0.99	0.24
	G‘‘ (Pa) 0.19 M Na^+	1.55/1.94/1.69	1.72	0.20
	η (mPa·s) 0.19 M Na^+	1062/970/1059	1031	52.27
	G‘ (Pa) 0.24 M Na^+	0.52/0.83/0.47	0.60	0.19
	G‘‘ (Pa) 0.24 M Na^+	1.40/2.09/1.45	1.64	0.39
	η (mPa·s) 0.24 M Na^+	449/430/390	423	30.12
	G‘ (Pa) 0.38 M Na^+	0.61/0.67/0.59	0.62	0.04
	G‘‘ (Pa) 0.38 M Na^+	2.12/1.13/1.99	1.75	0.54
	η (mPa·s) 0.38 M Na^+	487/469/481	479.3	9.17
	G‘ (Pa) 2.19 M Na^+	0.83/0.66/0.76	0.75	0.09
	G‘‘ (Pa) 2.19 M Na^+	1.84/1.04/1.60	1.50	0.41
	η (mPa·s) 2.19 M Na^+	521/602/570	564	40.80
	G‘ (Pa) 5.18 M Na^+	1.93/1.65/1.69	1.76	0.15
	G‘‘ (Pa) 5.18 M Na^+	2.52/2.17/1.90	2.19	0.31
	η (mPa·s) 5.18 M Na^+	790/818/813	807	14.93
**Figure 2C**	G‘ (Pa) 3.75 mM Ca^{2+}	0.92/1.31/0.85	1.03	0.25
	G‘‘ (Pa) 3.75 mM Ca^{2+}	1.30/2.22/1.10	1.54	0.60
	η (mPa·s) 3.75 mM Ca^{2+}	1042/875/1180	1031	152.7
	G‘ (Pa) 5.25 mM Ca^{2+}	0.78/0.40/0.60	0.58	0.19
	G‘‘ (Pa) 5.25 mM Ca^{2+}	1.21/0.80/1.22	1.07	0.24
	η (mPa·s) 5.25 mM Ca^{2+}	853/524/634	670.8	167.5
	G‘ (Pa) 8.78 mM Ca^{2+}	1.21/0.81/1.25	1.10	0.24
	G‘‘ (Pa) 8.78 mM Ca^{2+}	2.14/1.19/1.94	1.76	0.50
	η (mPa·s) 8.78 mM Ca^{2+}	1970/3031/2272	2423	546.7
	G‘ (Pa) 13.95 mM Ca^{2+}	1.43/1.31/2.48	1.74	0.64
	G‘‘ (Pa) 13.95 mM Ca^{2+}	2.12/1.26/4.01	2.40	1.34

	η (mPa·s) 13.95 mM Ca^{2+}	3468/2879/4575	3639	861.1
	G‘ (Pa) 24.16 mM Ca^{2+}	3.02/4.14/2.53	3.23	0.83
	G‘‘ (Pa) 24.16 mM Ca^{2+}	4.11/4.88/3.23	4.04	0.83
	η (mPa·s) 24.16 mM Ca^{2+}	8869/7914/6929	7902	970.0
	G‘ (Pa) 51.37 mM Ca^{2+}	2.68/2.91/3.85	3.16	0.62
	G‘‘ (Pa) 51.37 mM Ca^{2+}	2.59/2.01/3.16	2.57	0.58
	η (mPa·s) 51.37 mM Ca^{2+}	18971/11940/9924	13913	4749
	G‘ (Pa) 61.57 mM Ca^{2+}	0.91/0.84/1.04	0.93	0.10
	G‘‘ (Pa) 61.57 mM Ca^{2+}	1.55/0.89/1.38	1.27	0.34
	η (mPa·s) 61.75 mM Ca^{2+}	1083/1631/1302	1340	275.9
	G‘ (Pa) 78.58 mM Ca^{2+}	0.11/0.26/0.24	0.20	0.08
	G‘‘ (Pa) 78.58 mM Ca^{2+}	0.19/0.32/0.51	0.33	0.16
	η (mPa·s) 78.58 mM Ca^{2+}	408/542/385	445.7	84.79
	G‘ (Pa) 100.35 mM Ca^{2+}	0.57/0.24/0.45	0.43	0.17
	G‘‘ (Pa) 100.35 mM Ca^{2+}	0.37/0.41/1.01	0.58	0.36
	η (mPa·s) 100.35 mM Ca^{2+}	143.2/172.5/124.6	147.7	24.15
	G‘ (Pa) 150.01 mM Ca^{2+}	0.13/0.68/0.34	0.35	0.28
	G‘‘ (Pa) 150.01 mM Ca^{2+}	1.26/0.40/0.65	0.77	0.44
	η (mPa·s) 150.01 mM Ca^{2+}	243.8/182.5/207.2	210.4	30.84
**Figure 2D**	G‘ (Pa) 1.4 mM Mg^{2+}	0.92/1.31/0.85	1.03	0.25
	G‘‘ (Pa) 1.4 mM Mg^{2+}	1.30/2.22/1.10	1.54	0.60
	η (mPa·s) 1.4 mM Mg^{2+}	1042/875/1180	1031	152.7
	G‘ (Pa) 4.97 mM Mg^{2+}	5.71/4.14/3.90	4.58	0.98
	G‘‘ (Pa) 4.97 mM Mg^{2+}	5.32/4.50/4.26	4.69	0.56
	η (mPa·s) 4.97 mM Mg^{2+}	2048/1907/1002	1652	567.6
	G‘ (Pa) 8.75 mM Mg^{2+}	7.67/7.00/5.48	6.60	1.12
	G‘‘ (Pa) 8.75 mM Mg^{2+}	5.75/5.07/6.21	5.67	0.57
	η (mPa·s) 8.75 mM Mg^{2+}	1836/2196/1818	1950	213.2
	G‘ (Pa) 13.40 mM Mg^{2+}	7.21/5.92/10.92	8.02	2.60
	G‘‘ (Pa) 13.40 mM Mg^{2+}	9.05/7.66/10.68	9.13	1.15
	η (mPa·s) 13.40 mM Mg^{2+}	3730/1780/2581	2697	980.2
	G‘ (Pa) 24.0 mM Mg^{2+}	1.73/1.39/2.38	1.83	0.47
	G‘‘ (Pa) 24.0 mM Mg^{2+}	4.30/3.47/3.50	3.76	0.47
	η (mPa·s) 24.0 mM Mg^{2+}	578/550/1118	748.7	320.2
	G‘ (Pa) 49.4 mM Mg^{2+}	0.55/0.72/0.91	0.73	0.18
	G‘‘ (Pa) 49.4 mM Mg^{2+}	1.49/1.87/1.79	1.72	0.20
	η (mPa·s) 49.4 mM Mg^{2+}	715/200/412	442.3	258.8
	G‘ (Pa) 76.4 mM Mg^{2+}	0.95/0.90/0.77	0.88	0.09
	G‘‘ (Pa) 76.4 mM Mg^{2+}	1.87/1.52/1.23	1.54	0.32
	η (mPa·s) 76.4 mM Mg^{2+}	331/348/394	357.7	32.59
	G‘ (Pa) 101.4 mM Mg^{2+}	0.75/1.09/1.29	1.05	0.27
	G‘‘ (Pa) 101.4 mM Mg^{2+}	1.48/1.56/2.47	1.84	0.55
	η (mPa·s) 101.4 mM Mg^{2+}	470/331/143	314.7	164.1
	G‘ (Pa) 151.4 mM Mg^{2+}	1.32/2.81/1.13	1.32	0.92
	G‘‘ (Pa) 151.4 mM Mg^{2+}	2.19/2.81/2.23	2.41	0.35
	η (mPa·s) 151.4 mM Mg^{2+}	490/951/867	769	245.5
**Figure 2E**	G‘ (Pa) 0.0 mg/mL Lysozyme	1.08/0.71/1.15	0.99	0.24
	G‘‘ (Pa) 0.0 mg/mL Lysozyme	1.55/1.94/1.69	1.72	0.20
	η (mPa·s) 0.0 mg/mL Lysozyme	1062/970/1059	1031	52.27

	G‘ (Pa) 1.0 mg/mL Lysozyme	1.45/1.25/1.36	1.35	0.10
	G‘‘ (Pa) 1.0 mg/mL Lysozyme	2.87/2.34/2.50	2.60	0.27
	η (mPa·s) 1.0 mg/mL Lysozyme	1037/1659/1734	1476	382.6
	G‘ (Pa) 2.0 mg/mL Lysozyme	1.81/1.43/1.43	1.55	0.22
	G‘‘ (Pa) 2.0 mg/mL Lysozyme	3.08/2.64/2.76	2.83	0.23
	η (mPa·s) 2.0 mg/mL Lysozyme	1574/1243/1120	1313	234.8
	G‘ (Pa) 10.0 mg/mL Lysozyme	8.25/6.67/8.65	7.85	1.05
	G‘‘ (Pa) 10.0 mg/mL Lysozyme	8.13/6.99/7.60	7.57	0.57
	η (mPa·s) 10.0 mg/mL Lysozyme	36436/53704/56291	48818	10794
**Figure 2F**	G‘ (Pa) 1.21 mg/mL DNA	1.08/0.71/1.15	0.99	0.24
	G‘‘ (Pa) 1.21 mg/mL DNA	1.55/1.94/1.69	1.72	0.20
	η (mPa·s) 1.21 mg/mL DNA	1062/970/1059	1031	52.27
	G‘ (Pa) 5.0 mg/mL DNA	0.46/0.36/0.45	0.42	0.06
	G‘‘ (Pa) 5.0 mg/mL DNA	0.66/0.83/0.31	0.60	0.27
	η (mPa·s) 5.0 mg/mL DNA	626/335/355	438	162.5
	G‘ (Pa) 10.0 mg/mL DNA	0.52/0.35/0.41	0.43	0.09
	G‘‘ (Pa) 10.0 mg/mL DNA	0.59/1.06/0.80	0.81	0.24
	η (mPa·s) 10.0 mg/mL DNA	841/1037/857	911	108.8
	G‘ (Pa) 15.0 mg/mL DNA	1.26/0.96/1.01	1.06	0.16
	G‘‘ (Pa) 15.0 mg/mL DNA	1.66/1.13/1.41	1.40	0.27
	η (mPa·s) 15.0 mg/mL DNA	960/1637/1236	1287	340.4
	G‘ (Pa) 20.0 mg/mL DNA	1.03/1.35/1.17	1.18	0.16
	G‘‘ (Pa) 20.0 mg/mL DNA	1.16/1.88/1.67	1.57	0.37
	η (mPa·s) 20.0 mg/mL DNA	2290/1118/1645	1684	586.9

Table S1: *Storage modulus G’ (Pa) and loss modulus G’’ (Pa) at a frequency of 1.0 Hz respectively 6.28 rad/s and shear viscosity η (mPa·s) at a shear rate of 1.0* s^{-1} *are shown as individual values of the n=3 measurements, as well as the mean ± standard deviation (SD) of the data shown in Figure 1 and 3 as well as Figure S4, S5, S6, S7, S8, S9 and S10.*

2.6 Detailed analysis of the rheological data

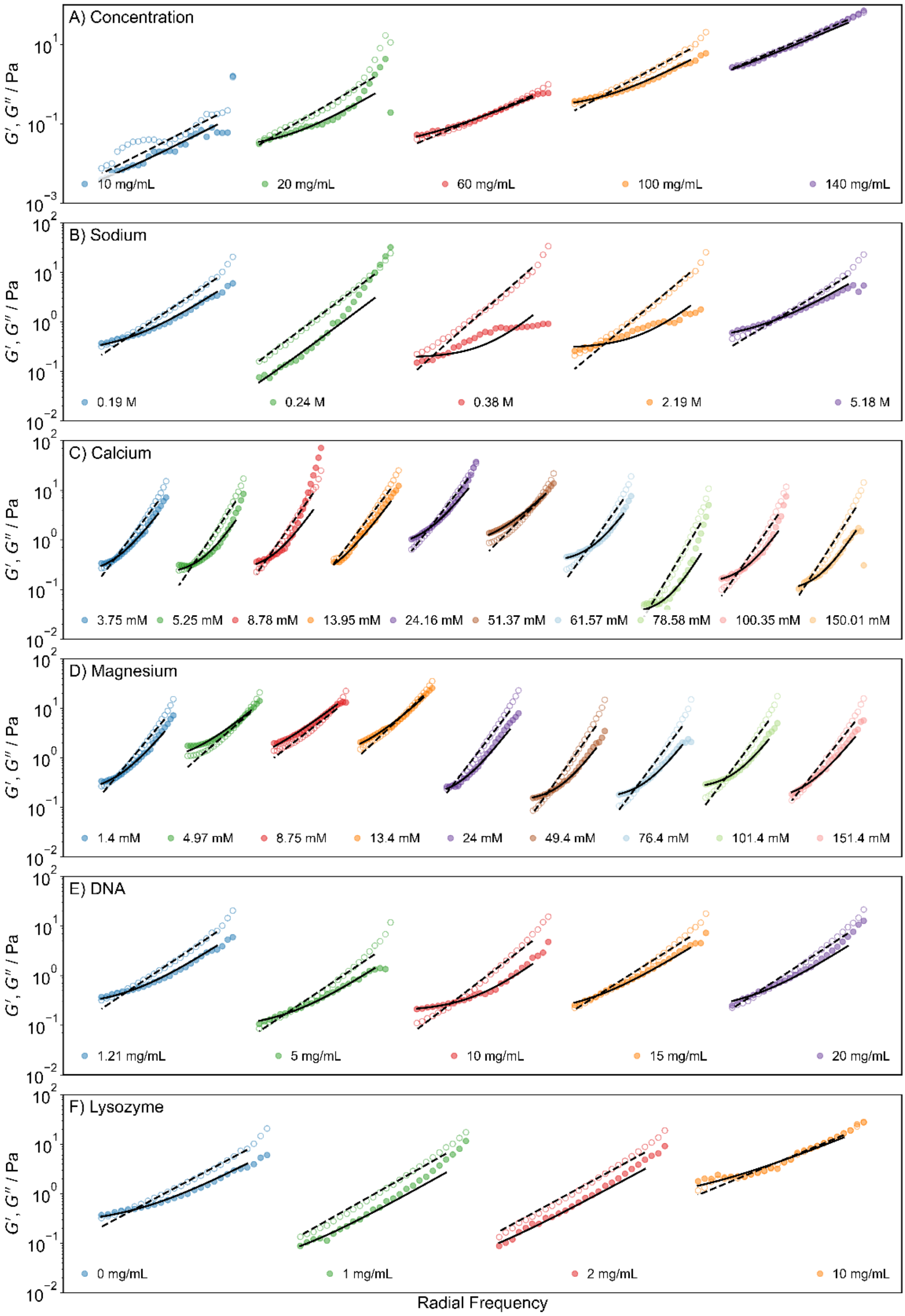

Figure S11: *Storage modulus G' (Pa) and loss modulus G'' (Pa) as function of angular frequency (shifted by* 10^3 *rad/s relative to each other); experimental data from rheological measurements (circles) fitted with the Fractional Kelvin-Voigt Model (black lines) for A) the*

five different concentrated BSM solutions, B) the 100 mg/mL BSM solutions with different concentrations of added sodium chloride, C) the 100 mg/mL BSM solutions with different concentrations of added calcium chloride, D) the 100 mg/mL BSM solutions with different concentrations of added magnesium chloride E) the 100 mg/mL BSM solutions with five different concentrations of added lysozyme, F) the 100 mg/mL BSM solutions with four different concentrations of added DNA. The angular frequency of the first sample in each series is in rad/s.

All frequency dependent G' and G'' data were fitted with a fractional Kelvin-Voigt model (FKVM), consisting of a spring and fractional spring-pot arranged in parallel. The model has three fit parameters: the spring's elastic modulus G the spring-pot's quasi-property η_α and the fractional exponent α. Only frequencies below 50 rad/s were considered for the fit.

The fit quality is generally rather good, despite the low number of parameters. For samples where the fit significantly deviates from the data, it is unclear whether this is due to problems of the fit or due to outliers in the data and/or measurement uncertainties. In some cases, the fit quality could be improved by using a second fractional spring-pot instead of a spring in the model. However, this would lead to an additional fit parameter and overfitting. Therefore, our simple FKVM model is a good compromise for the present data set, providing a reasonable description of the data with a minimal number of parameters.

The fitted FKVM parameters are discussed in detail in the main text.

2.7 Proteome analysis: Table of intensities measured for the peptides in BSM

We provide a detailed table of the intensities measured for the peptides in BSM of the three aliquots in a separate excel file. The table includes information about the following parameters for MUC19, BSM1 S1 and BSM1 S2: Sequence, Proteins, Leading razor protein, Start position, End position, Unique (Groups), Unique (Proteins), Identification type sample aliquot 1-3, Intensity sample aliquot 1-3 and LFQ intensity sample aliquot 1-3.

References

(1) Nisizawa, K.; Pigman, W. The composition and properties of the mucin clot from cattle submaxillary glands. *Arch Oral Biol* **1959**, *1*, 161-170. DOI: 10.1016/0003-9969(59)90008-1

(2) Schindelin, J.; Arganda-Carreras, I.; Frise, E.; Kaynig, V.; Longair, M.; Pietzsch, T.; Preibisch, S.; Rueden, C.; Saalfeld, S.; Schmid, B.; et al. Fiji: an open-source platform for biological-image analysis. *Nat Methods* **2012**, *9* (7), 676-682. DOI: 10.1038/nmeth.2019

(3) Frangakis, A. S. It's noisy out there! A review of denoising techniques in cryo-electron tomography. *J Struct Biol* **2021**, *213* (4), 107804. DOI: 10.1016/j.jsb.2021.107804

(4) Otsu, N. A Threshold Selection Method from Gray-Level Histograms. *IEEE Transactions on Systems, Man, and Cybernetics* **1979**, *9* (1), 62-66. DOI: 10.1109/TSMC.1979.4310076.

(5) Ruzyla, K. Characterization of Pore Space by Quantitative Image Analysis. *SPE Formation Evaluation* **1986**, *1* (04), 389-398. DOI: 10.2118/13133-PA (acccessed 11/23/2023).

(6) Budai, L.; Budai, M.; Pápay, Z.; Vilimi, Z.; Antal, I. Rheological Considerations of Pharmaceutical Formulations: Focus on Viscoelasticity. *Gels* **2023**, *9*, 469. DOI: 10.3390/gels9060469.

(7) Lafforgue, O.; Seyssiecq, I.; Poncet, S.; Favier, J. Rheological properties of synthetic mucus for airway clearance. *J Biomed Mater Res A* **2018**, *106* (2), 386-396. DOI: 10.1002/jbm.a.36251
